# Short tandem repeat polymorphism in the promoter region of cyclophilin 19B drives its transcriptional upregulation and contributes to drug resistance in the malaria parasite *Plasmodium falciparum*

**DOI:** 10.1371/journal.ppat.1011118

**Published:** 2023-01-25

**Authors:** Michal Kucharski, Grennady Wirjanata, Sourav Nayak, Josephine Boentoro, Jerzy Michal Dziekan, Christina Assisi, Rob W. van der Pluijm, Olivo Miotto, Sachel Mok, Arjen M. Dondorp, Zbynek Bozdech

**Affiliations:** 1 School of Biological Sciences, Nanyang Technological University, Singapore; 2 Mahidol-Oxford Tropical Medicine Research Unit, Faculty of Tropical Medicine, Mahidol University, Bangkok, Thailand; 3 Centre for Tropical Medicine and Global Health, Nuffield Department of Medicine, University of Oxford, Oxford, United Kingdom; 4 Center of Tropical Medicine and Travel Medicine, Department of Infectious Diseases, Academic Medical Center, University of Amsterdam, Amsterdam, The Netherlands; 5 Center for Malaria Therapeutics and Antimicrobial Resistance, Division of Infectious Diseases, Department of Medicine, Columbia University Irving Medical Center, New York, New York, United States of America; Weill Medical College of Cornell University, UNITED STATES

## Abstract

Resistance of the human malaria parasites, *Plasmodium falciparum*, to artemisinins is now fully established in Southeast Asia and is gradually emerging in Sub-Saharan Africa. Although nonsynonymous SNPs in the *pfk13* Kelch-repeat propeller (KREP) domain are clearly associated with artemisinin resistance, their functional relevance requires cooperation with other genetic factors/alterations of the *P*. *falciparum* genome, collectively referred to as genetic background. Here we provide experimental evidence that *P*. *falciparum* cyclophilin 19B (PfCYP19B) may represent one putative factor in this genetic background, contributing to artemisinin resistance via its increased expression. We show that overexpression of PfCYP19B *in vitro* drives limited but significant resistance to not only artemisinin but also piperaquine, an important partner drug in artemisinin-based combination therapies. We showed that PfCYP19B acts as a negative regulator of the integrated stress response (ISR) pathway by modulating levels of phosphorylated eIF2α (eIF2α-P). Curiously, artemisinin and piperaquine affect eIF2α-P in an inverse direction that in both cases can be modulated by PfCYP19B towards resistance. Here we also provide evidence that the upregulation of PfCYP19B in the drug-resistant parasites appears to be maintained by a short tandem repeat (SRT) sequence polymorphism in the gene’s promoter region. These results support a model that artemisinin (and other drugs) resistance mechanisms are complex genetic traits being contributed to by altered expression of multiple genes driven by genetic polymorphism at their promoter regions.

## Author summary

The malaria parasite is a complex eukaryotic pathogen able to mount resistance quickly to any given drug therapy. Frequently, resistance mechanisms depend on not only one but the cooperation of several genetic elements simultaneously to be retained in the population. These interactions remain currently very rarely studied by modern malaria research. Here we have explored in more detail one of the putative genetic background elements–cyclophilin 19B and its effect on drug resistance in the malaria parasite. We have shown that parasites overexpressing this protein showed higher tolerance to two important antimalarial drugs–dihydroartemisinin and piperaquine. This tolerance likely involves an important eukaryotic stress response and signaling pathway known as Integrated Stress Response. We have also uncovered the connection between cyclophilin 19B expression level and a genomic DNA sequence polymorphism in the short tandem repeat AT-rich region located in its gene promoter area. This study provides an important framework for further exploration of the function of abundant AT-rich elements present in the *Plasmodium falciparum* genome and its relation to differential gene expression, phenotypic variation, and eventually selection of drug resistance.

## Introduction

The progressively decreasing efficacy of the artemisinin-based combination therapies (ACT) in the treatment of *Plasmodium falciparum*-caused malaria infections is being reported in the Greater Mekong Subregion (GMS) over the last decade [1–3]. This is highly worrisome given that ACTs represent the first-line therapy for malaria throughout the entire malaria-endemic world, with over 600,000 deaths and more than 200 million cases reported in the year 2020 alone [4]. These ACT failures can be traced to the rise of artemisinin resistance in eastern Cambodia between 2005–2009 and its progressive spread throughout the entire GMS over the following decade [1,2,5]. The artemisinin resistance phenotype is strongly linked with nonsynonymous single nucleotide polymorphisms (nsSNP) in the *P*. *falciparum pfk13* gene and is clinically manifested by a prolonged parasite clearance half-life (PC1/2) after ACT administration, leading to persistent parasitemia for up to 6–7 days (compared to 2–3 days in susceptible infections) [6,7]. This leads to a significant increase of parasite load throughout the treatment course, which promotes further refinement of not only the putative artemisinin resistance mechanism *per se* but also (re)selection of resistance to the ACT partner drugs. This includes resistance to mefloquine (MFQ), the key partner drug in the Artesunate/MFQ-based ACT that is associated with an increased copy number of *pfmdr1* [8,9]. Similarly, resistance to piperaquine (PPQ) selected by the Dihydroartemisinin (DHA)/PPQ-based ACT [10–13] is associated with an increased copy number of plasmepsin 2/3 locus [14,15] and several SNPs in the *pfcrt* gene [16–19]. Taken together, the diminishing effectiveness of ACTs that is currently ongoing in the GMS likely reflects the changing genetic makeup of the *P*. *falciparum* parasite population driven by constant drug selection pressure [16,20].

This selection process is mirrored in the evolutionary pattern by which each of the known resistance alleles emerged and spread over the last decade. Initially, more than 30 nsSNP of *pfk13* emerged throughout the GMS in the first decade of the 21st century independently [1,6,20,21]. Between 2010–2017, however, two major alleles (C580Y and F446I) underwent a strong selective sweep in the eastern GMS and northwestern Myanmar, respectively [16]. Although these SNPs do not confer the strongest artemisinin resistance phenotype *in vitro*, their selective sweep likely balances fitness deficits in the context of genetic background of the parasite populations within the eastern and western GMS (eGMS and wGMS, respectively). Given that artemisinins are the invariant component of the ACT applied throughout the GMS, the *pfk13* SNP selective sweep is most likely a direct result of the drug’s selective pressure in both regions. On the other hand, amplification of *pfmdr1*, the original resistance marker of MFQ monotherapy detected along the western border of Thailand, emerged as a result of intense sustained administration of artesunate/MFQ ACT between 2000–2011 [8]. Likewise, the high frequencies of plasmepsin 2/3 amplification and *pfcrt* SNPs in the eGMS were driven by the deployment of PPQ as the main ACT partner drug used in this region over the last decade [14,15]. This indicates that the genetic flux of GMS *P*. *falciparum* malaria parasite population is strongly influenced by the choice of the administrated chemotherapeutics, driving multiple alleles of drug resistance to individual components of the ACTs in parallel.

Even though the significance of the few above-mentioned genetic polymorphisms for drug resistance is well documented, there is mounting evidence suggesting that each of these functions in conjunction with a specific genetic background. First, the *P*. *falciparum* population in the epicenter of artemisinin resistance—the western provinces of Cambodia, is composed of several founder subpopulations [22–24] carrying specific polymorphisms covariant with the *pfk13* SNPs [25]. Second, the introduction of *pfk13* SNPs drives a profoundly stronger artemisinin resistance phenotype in *in vitro* cultured parasites with “Cambodian” origin compared to those with unrelated genetic backgrounds [7]. The existence of genetic background is also underlined by the recent report describing artemisinin-resistant parasites in Africa [26,27]. Here, two *pfk13* mutations (A675V and C469Y) were found to be associated with clinical resistance to artesunate. However, a follow-up study with CRISPR-edited parasites showed that parasites harboring these mutations only have marginally reduced susceptibility to DHA compared to their wild type counterparts and parasites harboring R539T mutation commonly found in Southeast Asia [28]. In case of PPQ, episomal overexpression of plasmepsin 2/3 failed to yield resistance *in vitro* [29] but freshly adapted Cambodian *P*. *falciparum* isolates with plasmepsin 2/3 amplification exhibit profoundly decreased PPQ susceptibilities [14,15]. Similar to *pfk13* nsSNP, inconsequential amplification of plasmepsin 2/3 has been reported in several *in vivo P*. *falciparum* populations, suggesting a need for specific genetic background for PPQ resistance as well [30]. Altogether, this suggests that the ACT-related drug resistance alleles operate in the context of other genetic variations in order to exert their mechanistic effect of decreased drug susceptibility that in the end leads to treatment failure. A comprehensive understanding of this genetic background is crucial for further management of the currently available ACTs but also for the design of new treatments, such as the introduction of triple combination therapy (TACT) that might reduce the onset of malaria drug resistance around the world [3].

In our recent transcriptomic analysis, we uncovered a number of putative genes constituting the drug-resistance genetic background, by defining the artemisinin resistance-associated transcriptional profile (ARTP) [31]. The ARTP consists of at least 156 genes whose altered expression has the potential to contribute to the overall drug resistance phenotype(s). Along with genes involved in protein folding and the IRS, the ARTP also highlighted differential expression of hemoglobin digestion and protein synthesis/degradation, both of which were recently linked with artemisinin resistance in *pfk13* mutants [32–34]. Here in this study, we provide experimental evidence that overexpression of one of the ARTP genes, *P*. *falciparum* cyclophilin 19B (PfCYP19B; PF3D7_1115600), can drive a limited but significant level of resistance to DHA and PPQ. This is mediated by the PfCYP19B biological function in protein folding that is linked with signaling of the parasite’s integrated stress response (ISR) via phosphorylation of eIF2α (eIF2α-P). Both artemisinin and piperaquine can affect levels of eIF2α-P, albeit in inverse direction, which suggests a certain level of an overlap between their modes of action (MOA). Here we show that the upregulation of PfCYP19B resulting in suppression of eIF2α-P can drive resistance to both drugs. The results presented in this study provide first evidence for an intriguing model in which malaria parasites fine-tune their gene expression levels via STR-driven polymorphisms to create suitable physiological profiles that underline key complex phenotypic traits (e.g., drug resistance).

## Results

### PfCYP19B is consistently upregulated in the artemisinin-resistant parasites *in vivo*

**Fig 1:**
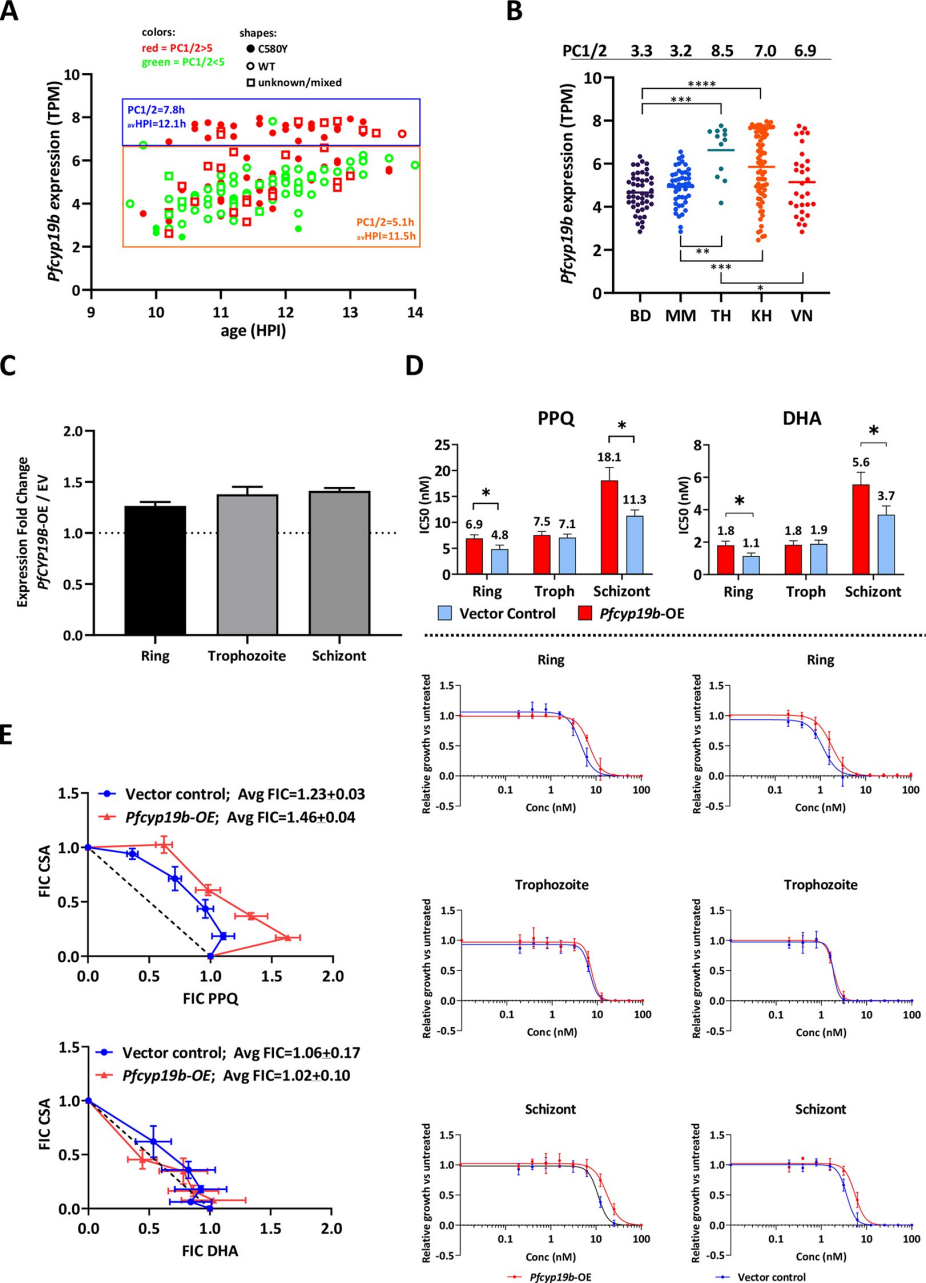
PfCYP19B upregulation plays role in modulating antimalarial drug response. (A) *Pfcyp19b* RNA-Seq expression values derived from samples collected during the TRACII study at patient’s admission for the treatment (2015–2018) (n = 173) [31]. Expression values (transcripts per million, TPM) were projected against parasites’ age (HPI). The steady increase in the expression of *pfcyp19b* can be seen with the parasite’s age. Resistant (PC1/2 > 5) and sensitive (PC1/2 < 5) parasites are shown in red and green respectively. Shapes in the scatter plot represent the type of *pfk13* mutation in each sample (C580Y mutant–full dots, wild type (WT)–empty circles, unknown mutation type or mixed WT/C580Y populations–empty squares). Two significantly distinct groups of parasites can be observed based on *pfcyp19b* expression levels; the first group (upper blue rectangle blue) expressing higher levels of *pfcyp19b* transcripts regardless of parasite’s age, second group (lower orange rectangle) expressing lower levels of *pfcyp19b* (*p* = 2.8E-72, unpaired two-tailed t-test). There is also a significant difference in PC1/2 (*p* = 1.7E-13, unpaired two-tailed t-test) between these two groups as shown in the graph. Parasites within the orange rectangle exhibit lower PC1/2 values and mostly are of wild type *pfk13* background (B) *Pfcyp19b* expression levels by country in TRACII study (BD–Bangladesh, MM- Myanmar, TH–Thailand, KH–Cambodia, VN–Vietnam). Average PC1/2 are indicated above the graphs showing tight positive linear correlation between mean *pfcyp19b* expression values (per each site) and the mean PC1/2 (PCC = 0.9). Asterixes indicate *p*-values of significance in *pfcyp19b* expression between the sites based on Brown-Forsythe ANOVA test (* *p* < 0.05, ** *p* < 0.01, *** *p* < 0.001, **** *p* < 0.0001) (C) Fold change increase of total PfCYP19B protein expression in 3D7 *PfCYP19B*-OE strain compared to Empty Vector control in three distinct developmental stages of the parasite as measured by densitometry. Error bars indicate standard deviation (n = 2) (D) IC_50_ values from piperaquine (PPQ; left) and dihydroartemisinin (DHA; right) drug assays performed on 3D7 *Pfcyp19b-*OE line compared to empty Vector controls (top panel) and their respective dose-response curves (bottom panel). Assays were performed on 3 distinct developmental stages (i.e. ring at 4 HPI, trophozoite at 20 HPI, and schizont at 35 HPI). The numbers above bars represent the average IC_50_ values whereas error bars indicate standard deviation of the IC_50_ from each treatment conducted in 3 independent biological replicates. Asterixes indicate statistical significance (* *p* < 0.05) based on unpaired, two-tailed heteroscedastic *t*-test (E) Modified fixed-ratio isobologram graphs showing drug interaction between PPQ and CsA (top) and DHA and CsA (bottom). Average fractional inhibitory concentration values were derived from three independent biological replicates.

The main goal of this study was to investigate the role of PfCYP19B in the multifaceted mechanism of artemisinin resistance in the *P*. *falciparum*. This was motivated by our previous population transcriptomics analyses in which transcription of *pfcyp19b* was consistently and significantly upregulated in artemisinin-resistant parasites. These include two epidemiological surveys carried out across multiple sites of the GMS between 2011–2013 (TRACI) and 2015–2018 (TRACII) in both of which, increased *pfcyp19b* transcript levels were positively correlated with the prolonged parasite clearance half-life (PC1/2) in non-severe malaria patients treated with ACTs (**S1 Fig**) [31,35]. This correlation is particularly well illustrated by the RNA sequencing measurements (RNA-Seq) of a subset (n = 173) of TRACII samples (**Fig 1A**). Overall, *pfcyp19b* showed elevated mRNA levels in the slow-clearing parasites (PC1/2 > 5, Transcripts per Million (TPM) _mean_ = 5.7, red marks) compared to fast-clearing parasites (PC1/2 < 5, TPM_mean_ = 4.7, green marks) (*p* = 3.46E-06, two-tailed unpaired t-test). Throughout the age projection (Hours Post Invasion, HPI), we can separate the parasite samples into two groups including parasites with persistently high *pfcyp19b* expression (TPM_mean_ = 7.5, upper blue rectangle) and parasites with low *pfcyp19b* expression (TPM_mean_ = 4.6, lower orange rectangle) (**Fig 1A**). This accounts for 1.6-fold change in the average *pfcyp19b* transcript levels between the two groups (p = 2.8E-72). Crucially, the high-expressing parasite group consisted almost exclusively of artemisinin-resistant parasites (PC1/2 > 5) from which 78% carried C580Y (**Fig 1A,** full dots/marks). This contrasts the low expression group that consisted mainly of the susceptible parasites with only 32% of these carrying C580Y (**Fig 1A,** empty circles/marks). However, the increased expression values of *pfcyp19b* in the resistant parasites were particularly pronounced in the early stages (HPI < 11). Curiously, the basal-level, intra-erythrocytic developmental cycle (IDC) transcription profile of *pfcyp19b* is characterized by low mRNA abundance in the early ring stages [36]. This suggests that the observed overexpression of *pfcyp19b* in artemisinin-resistant parasites reflects its transcriptional de-repression during its minimal IDC expression. This is consistent with transcriptomic results of *in vitro* generated artemisinin-resistant parasites [37]. There we observed 2-fold *pfcyp19b* upregulation at the late schizont to early rings transition stage (48 HPI / 0 HPI) in the isogenic Cam3.II R539T Cambodian strain compared to Cam3.II wild type (average *log2* expression increase in 0HPI = 0.85, in 48HPI = 0.98) (**S2 Fig**). The early ring *pfcyp19b* upregulation coincides with the expected stage specificity of the *Pfk13*-driven artemisinin resistance measured by the *in vitro* ring survival assay (RSA) (0–3 HPI) [38]. From the population perspective, the transcriptional upregulation of *pfcyp19b* was overrepresented in the TRACII sites with higher occurrence of artemisinin resistance (PC1/2 > 5) including Thailand (TH), Cambodia (KH), and southern Vietnam (VN) (**Fig 1B**) (mean *pfcyp19b* expression levels (in TPM) to mean PC1/2 Pearson correlation coefficient (PCC) = 0.9). In contrast, no *pfcyp19b* upregulation was observed in Myanmar (MM) and Bangladesh (BD) sites, where no significant levels of artemisinin resistance have been reported at the time of this epidemiological study [19,31].

### PfCYP19B mediates limited but significant resistance of *P*. *falciparum* to artemisinin and piperaquine *in vitro* via its protein folding function

Next, we wished to investigate whether the increased expression of PfCYP19B could contribute to the *P*. *falciparum* resistance phenotype and, if so, what is the putative mechanism by which this protein exerts its biological effect. Previously reported large-scale essentiality screen identified two exonic insertions of the PiggyBac-based transposon into the *pfcyp19b* locus, initially suggesting its dispensability for the *P*. *falciparum* IDC growth [39]. Hence, we attempted to generate a complete *pfcyp19b* deletion strain using the homologous recombination strategy in the 3D7 parasite strain (see Materials and Methods and **S10 Fig**). However, we were unable to generate such a deletion transgenic cell line indicating that PfCYP19B may be at least to some degree indispensable. Indeed, the PiggyBac insertions were observed only at the extreme 3’ end of the *pfcyp19b* transcript leaving the majority of the open reading frame intact which could still yield an active protein product. Not being able to resolve the essentiality of PfCYP19B at this point, instead, we generated a *P*. *falciparum* transgenic parasite line by introducing an additional *pfcyp19b* copy to 3D7 laboratory strain in addition to the parasite’s endogenous gene copy by using episomal blasticidin-based titratable system [40]. We have achieved PfCYP19B protein overexpression across the IDC (**S3 Fig**) with 1.27, 1.38, and 1.41-fold higher protein levels in rings, trophozoites, and schizonts, respectively (**Figs 1C and S3**). Using this PfCYP19B overexpressing parasite line (*Pfcyp19b*-OE), we carried out drug sensitivity assays where the cells were treated by a drug at a particular IDC stage (ring 4 HPI, trophozoite 20 HPI, and schizont 35 HPI) and survival was monitored in the next invasion cycle under the sustained drug pressure. Crucially, *Pfcyp19b*-OE exhibited decreased sensitivity to both dihydroartemisinin (DHA) and piperaquine (PPQ) when the parasites were treated at the ring and schizont stages. For the schizont stage, we observed, 1.6-fold (*p* = 0.013) and 1.5-fold (*p* = 0.025) increases in 50% inhibitory concentrations (IC_50_) of *Pfcyp19b*-OE for PPQ and DHA, respectively, compared to the control cell line transfected with the identical transfection vector without *pfcyp19b* (empty Vector Control—EV) (**Fig 1D**). Similarly, 1.4-fold (*p* = 0.026) and 1.6-fold (*p* = 0.024) increases in IC_50_ were detected for *Pfcyp19b*-OE when the cells were treated at the ring stage with PPQ and DHA, respectively **(Fig 1D)**. Overexpression of PfCYP19B did not mediate increased resistance to PPQ and DHA at the trophozoite stage **(Fig 1D)**. Although above IC_50_ assays interestingly show altered drug sensitivity in PfCYP19B overexpressing lines, *Pfcyp19b*-OE did not show increased resistance to artemisinin in the ring survival assay (RSA) between 0–3 HPI, a golden standard for measuring drug’s sensitivity (**S4 Fig**). Indeed, while the increased IC_50_ values of the *Pfcyp19b*-OE demonstrate the ability of this protein to confer resistance to both DHA and PPQ, the resistance phenotypes differ from those in culture-adapted field strains measured by RSA and piperaquine survival assays [38,41,42]. This is likely due to the episomally-expressed PfCYP19B transitional profile that differs from the endogenous allele. Nevertheless, the increased resistance of the ring and schizont stages still coincides with the transcriptional profile of *pfcyp19b* with its mRNA peak levels in the late schizont stage and its protein products persisting through schizogony and invasion to early ring stages [36,37,43]. Most importantly these results indicate that PfCYP19B can mediate resistance to artemisinins and PPQ in a highly similar fashion which suggests that these two drugs share their MOAs and as such can select for identical mediators of resistance (such as PfCYP19B). Indeed, the upregulation of PfCYP19B in the GMS parasites coincides with the wide use of DHA/PPQ as the main combination therapy in the second decade of the 20th century [35,44].

Analogous to other cyclophilins, PfCYP19B is a peptidyl-prolyl cis-trans isomerase presumably mediating a broad range of functionalities associated with protein folding. As such, PfCYP19B can be inhibited by Cyclosporin A (CsA) that was shown to block the peptidyl-prolyl cis-trans isomerase active site directly [43,45]. In *P*. *falciparum*, CsA-mediated inhibition of cyclophilins was previously shown to suppress phosphatase activity of calcineurin by forming a calcineurin-cyclophilin-CsA complex which leads to inhibition of intraerythrocytic growth [46]. Indeed, CsA can affect the overall sensitivities of *P*. *falciparum* to PPQ indicating that cyclophilin-like activities overlap with its MOA (**S5 Fig**). Specifically, the continuous presence of 80nM of CsA (25% of CsA IC_50_) throughout the ring-initiated drug assay resulted in statistically significant changes of IC_50_ values for PPQ but not for amodiaquine (AQ), chloroquine (CQ), pyrimethamine (PYR), and DHA (**S5 Fig**). Accordingly, there was a strong antagonistic interaction between CsA and PPQ when tested by isobologram-based analyses which contrasts solely additive interaction between CsA and DHA (**Fig 1E**). Crucially, overexpression of PfCYP19B deepens the CsA-PPQ antagonisms which is indicated by the statistically significant increase in FIC index (*p* = 0.02, unpaired two-tailed t-test) in *Pfcyp19b*-OE (FICs = 1.46±0.04) compared to the empty Vector control line (FICs = 1.23±0.03) (**Fig 1E**). This strongly suggests that PfCYP19B plays a considerable role in the interaction between the CsA and PPQ cytotoxic activities in *P*. *falciparum*. It is also important to note that overexpression of PfCYP19B did not alter parasite sensitivities to CsA alone (**S6 Fig**). This is likely due to the fact that PfCYP19B plays only a minor role in the CsA MOA and any difference can be seen solely when parasites were subjected to the stress of additional drug (e.g. CsA and PPQ). Moreover, CsA also affected the sensitivities of *P*. *falciparum* to MFQ and atovaquone (ATQ), albeit in the opposite direction compared to PPQ (**S5 Fig**). Currently, nothing is known about the relationship between the MFQ and ATQ modes of action and the effects of CsA (presumably affecting protein folding). Although beyond the scope of this study, these results might suggest further explorations in this area, and their inverse relationship indicate a different mechanism of their interactions compared to PPQ.

### PfCYP19B functions as a negative regulator of the phosphorylated Eukaryotic translation initiation factor 2α (eIF2α)

**Fig 2:**
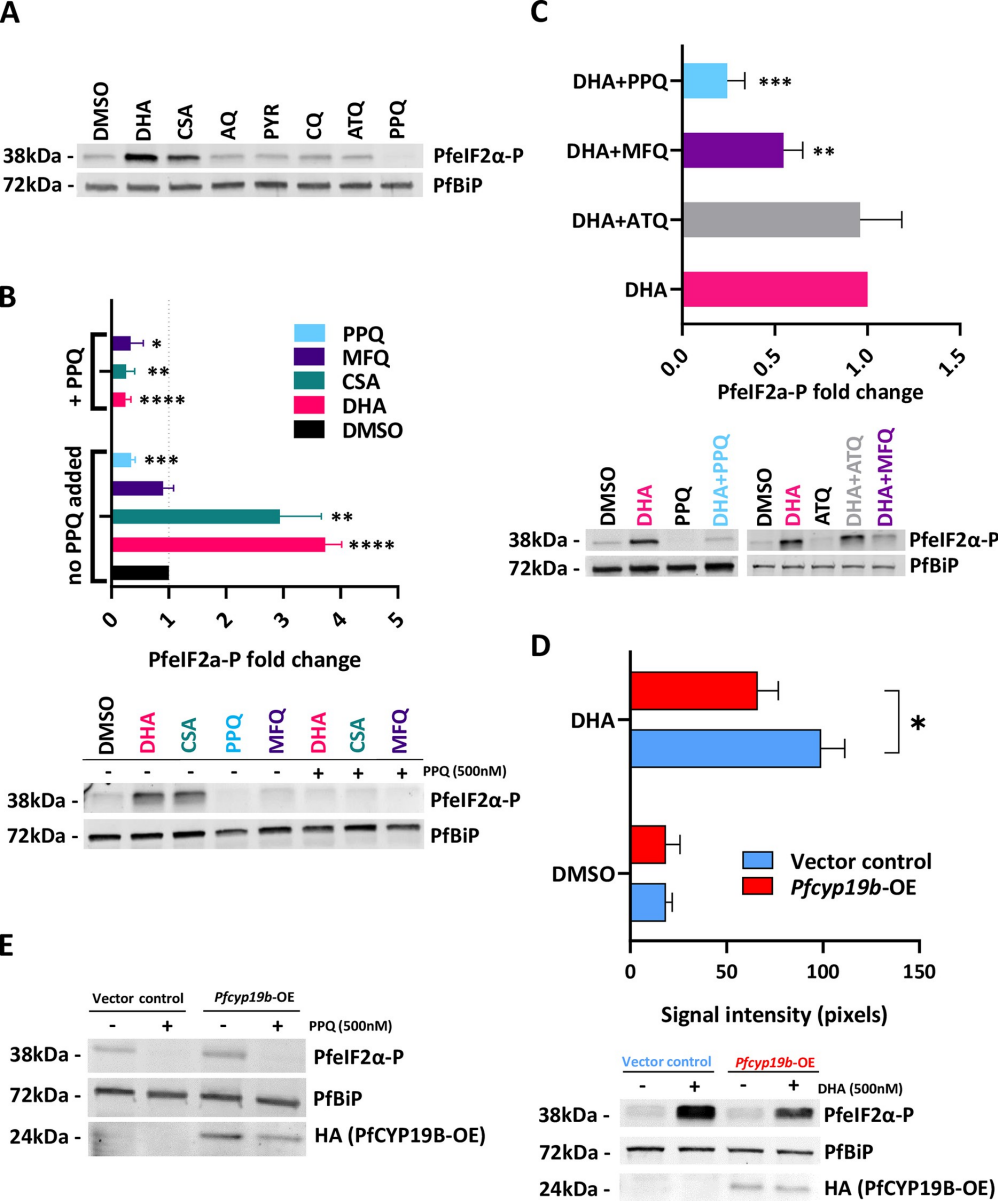
PfCYP19B affects the parasite’s drug sensitivity and acts as a negative regulator of PfeIF2α-P levels. (A) Western blot images showing a change in levels of PfeIF2α-P after 90 min treatment of 3D7 parasites (n = 1) with several distinctive antimalarial drugs in concentrations exceeding several folds their respective IC_50_ values (CsA– 4 μM, DHA– 0.5 μM, PPQ– 0.5 μM, ATQ– 0.5 μM, PYR– 1 μM, CQ– 1 μM) (B) Western blot images (below) illustrating the effect of 90 min 0.5 μM PPQ co-treatment with various high-dose antimalarial drugs on the PfeIF2α-P levels (CsA– 4 μM, DHA– 0.5 μM, MFQ– 2 μM, PPQ– 0.5 μM). The bar graph (above) shows fold changes of PfeIF2α-P after treatment with a single drug (no additional PPQ added) compared to DMSO control treatment with significant fold change to DMSO indicated by asterixes. The top part of the bar graph illustrates PfeIF2α-P fold change after additional 0.5 μM PPQ supplementation compared to PfeIF2α-P levels induced by each corresponding drug alone with significant fold change to its respective single-drug treatment indicated by asterixes. There was marked suppression of induction of PfeIF2α-P levels in all tested drugs after adding PPQ to each drug. Significant differences in PfeIF2α-P are indicated by asterixes (C) Western blot images (below) illustrating similar suppressive effect of two closely related aminoquinolines (MFQ, PPQ) and control non-aminoquinoline drug atovaquone (ATQ) on PfeIF2α-P induction. Graph (above) shows relative PfeIF2α-P values of co-treatments to PfeIF2α-P values induced by DHA treatment alone. 0.5 μM PPQ had the strongest suppressive effect on the PfeIF2α-P levels, followed by 2 μM MFQ. 2 μM ATQ had no suppressive effect on the induction of PfeIF2α-P levels caused by DHA treatment (D) Western blot analysis showing alleviating effect of PfCYP19B overexpression on PfeIF2α-P levels after ER stress induced by 90 min 0.5 μM DHA treatment. The experiment was performed on early ring stage parasites episomally overexpressing PfCYP19B (*Pfcyp19b*-OE) and empty Vector control line (E) Western blot analysis showing no measurable effect of PfCYP19B overexpression on PfeIF2α-P levels after 90 min 0.5 μM PPQ treatment. The experiment was performed on early ring stage *Pfcyp19b*-OE parasites and empty vector control (n = 1). All experiments shown in the figure were performed in 3 independent biological replicates unless indicated otherwise. Stars in the graphs indicate statistical significance based on unpaired two-tailed heteroscedastic t-test (* *p* < 0.05, ** *p* < 0.01, *** *p* < 0.001, **** *p* < 0.0001). All values shown are mean values with SD indicated by whiskers. All values were normalized to PfBiP internal loading control.

Similar to higher eukaryotes, *P*. *falciparum* utilizes phosphorylation of eIF2α as a key signaling factor in the Integrated Stress Response (ISR) by which the cells respond to a range of cellular stresses including proteotoxicity, ER stress, accumulation of misfolded proteins, starvations, heat shock or heme deprivation [47–49]. In this process, phosphorylation of eIF2α (eIF2α-P) is believed to mediate the downregulation of global protein synthesis on one side and the upregulation of specific genes countering the stress effects on the other [50,51]. Cyclophilin takes part in the ISR by assisting in protein folding, preventing their aggregation in the ER, and thus mitigating proteotoxicity [52,53]. Indeed, modes of action of ACT components including both artemisinins and PPQ, are known to invoke (some form of) proteotoxicity and cellular stresses such as oxidative damage [34,35,54]. Here we wished to investigate the role of PfCYP19B in the induction of PfeIF2α-P as a putative regulatory element of the ISR and ER stress in P. *falciparum* [33]. For that, we first investigated the effect of a spectrum of antimalarial compounds on the levels of eIF2α-P using western blot analyses with *P*. *falciparum* 3D7 *in vitro* culture (**Fig 2A**). Specifically, exposure of the ring stage parasites (10 HPI) to 4 μM of CsA and 500 nM DHA for 90 minutes resulted in elevated eIF2α-P levels; the latter being described previously [47]. This effect appears highly specific to the artemisinin derivatives and cyclosporines as neither of the other tested antimalarial drugs such as CQ, AQ, PYR, and ATQ affected the eIF2α-P levels (**Fig 2A**). Surprisingly, 90 minutes treatment with 500 nM PPQ exerted the opposite effect, eliminating even the baseline levels of eIF2α-P. PPQ is also able to suppress the DHA and CsA-induced eIF2α-P, bringing its levels even below the baseline (**Fig 2B**). This suppression is highly potent and dynamic as even 5 nM of PPQ (less than a fifth of its estimated IC_50_) can significantly decrease the levels of eIF2α-P (**S7 Fig**). Moreover, simultaneous treatment of *P*. *falciparum* with DHA/PPQ yielded significant suppressions of eIF2α-P despite the strong inducing effect of DHA alone (**Fig 2B and 2C**). Curiously, MFQ could also suppress eIF2α-P although to a lesser degree while ATQ had no effect on eIF2α-P neither as a single drug nor in combination with DHA (**Fig 2C**). These results suggest that the cytotoxic effect of both PPQ and DHA involve cellular stresses affecting the key component of ISR—eIF2α-P; albeit in the opposite manner. This further supports our observation that these two antimalarials share their MOA and, as such components of their resistance mechanisms including PfCYP19B.

Going further, we utilized the *Pfcyp19b*-OE line to investigate the potential of PfCYP19B interaction in eIF2α-P signaling. Indeed, while the elevated expression of PfCYP19B had no effect on the basal eIF2α-P levels, we observed significant suppression of eIF2α-P levels under DHA treatment (**Fig 2D**). Essentially, exposures of the EV line to 500nM of DHA for 90 min led to 5.3-fold increase of PfeIF2α-P, which in *Pfcyp19b*-OE was reduced to 3.6-fold corresponding to a 1.7-fold reduction (*p* = 0.025, two-tailed unpaired t-test). We observed similar but reduced effect with another inducer of PfeIF2α-P and the inhibitor of PfCYP19B –Cyclosporin A. We obtained a 3.1-fold increase in phosphorylation levels in *Pfcyp19b*-OE compared to a higher 3.8-fold increase in Empty Vector control (n = 1) (**S8 Fig**). No effect of PfCYP19B overexpression on the eIF2α-P could be observed after PPQ treatment likely due to its levels already nearing the background values (**Fig 2E**). Altogether, this suggests that PfCYP19B acts as a negative regulator of the stress-induced eIF2α-P levels presumably via its protein folding biological function. As a result PfCYP19B can play a role in *P*. *falciparum* resistance to both DHA and PPQ via its peptidyl-prolyl cis-trans isomerase activity.

### Cellular localization and protein partner interactions of PfCYP19B point to its ER-stress-related function

**Fig 3:**
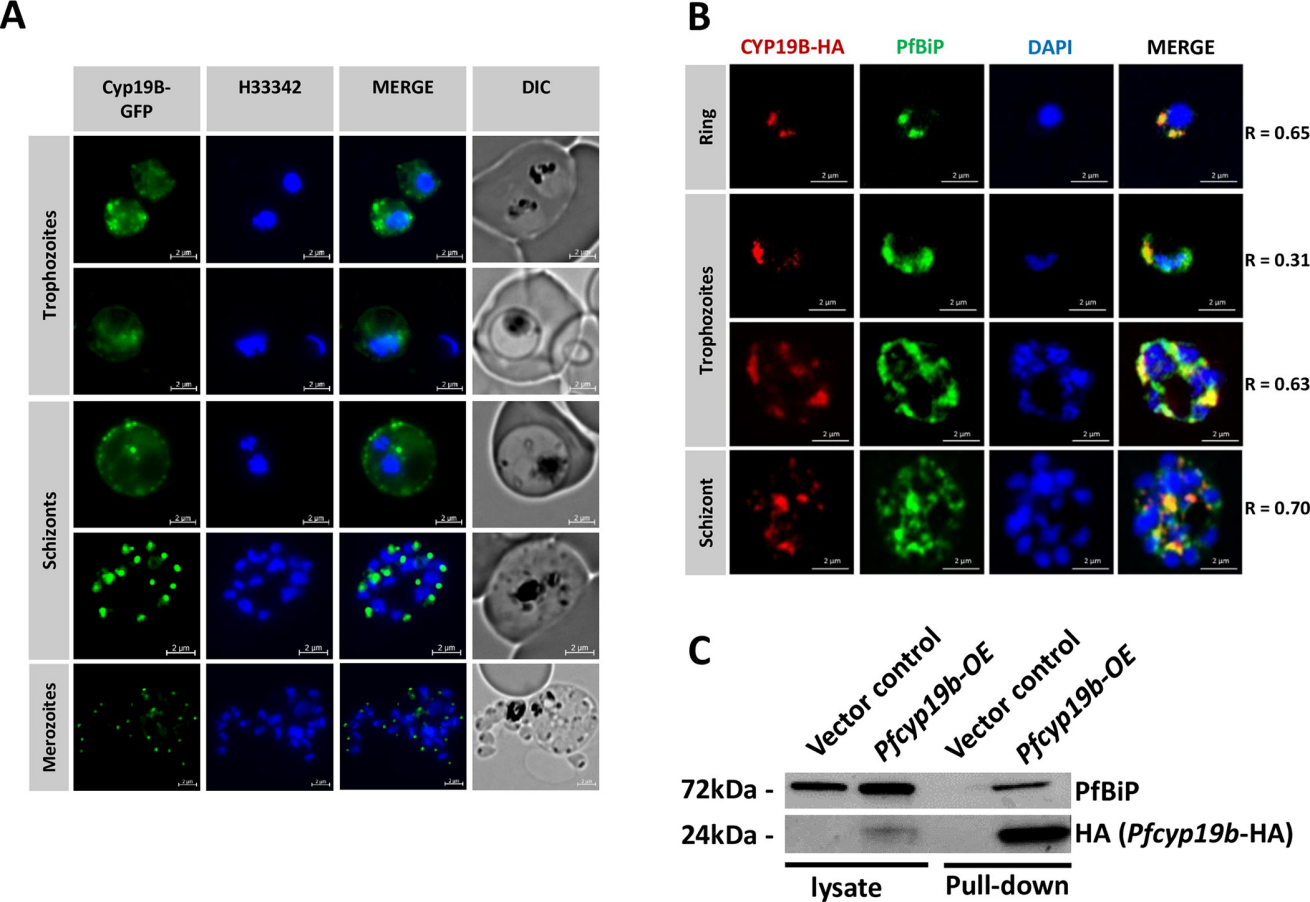
Cellular localization and protein partner interactions of PfCYP19B point to its ER-stress-related function. A) Live cell imaging of *P*. *falciparum* 3D7 parasite line with endogenously tagged PfCYP19B (green) counterstained with Hoechst33342 (blue). (B) Immunofluorescence microscopy of cells overexpressing PfCYP19B (red) with the ER marker PfBiP (green), counterstained with DAPI. Pearson’s colocalization coefficient for PfCYP19B and PfBiP in each image is shown on the right. (C) Western blot showing successful pull-down of PfBiP using episomally expressed HA-tagged PfCYP19B. An empty Vector control line was used as a negative control.

To study the cellular localization of PfCYP19B, we generated a GFP/FKBP-tagged PfCYP19B strain based on the *P*. *falciparum* 3D7 line (*Pfcyp19b*-GFP). Live cell imaging was able to detect PfCYP19B only in the second half of the IDC—trophozoite through schizont stages, in agreement with its transcription profile [36]. Here PfCYP19B localizes to the parasite cytoplasm with a punctate staining pattern (**Fig 3A**). In segmented schizonts, however, PfCYP19B localizes to distinct loci in the gradually maturing merozoites that persists after schizont rupture. The PfCYP19B signal is localized to the blunt end of the merozoite, with the nucleus situated between it and the apical protrusion (**Fig 3A**). The predominant structural feature of PfCYP19B is its peptidyl-prolyl cis-trans isomerase domain, which links this protein functionally with protein folding and also acts as a molecular chaperone [55,56]. Based on its evolutionary conservation, PfCYP19B is predicted to interact with *P*. *falciparum* Binding Immunoglobulin Protein homologue (PF3D7_0917900, PfBiP or PfGRP78) that is also known to facilitate/regulate protein folding in the ER [57–60]. To study this, we conducted immunofluorescence microscopy to determine the colocalization pattern of PfCYP19B and PfBiP. Our initial attempts to use the GFP/FKBP-tagged *P*. *falciparum* line with an anti-FKBP antibody failed to yield a good signal-to-noise ratio and therefore, we attempted immunofluorescence microscopy on the *Pfcyp19b*-OE strain instead. Indeed, indirect immunofluorescence assay (IFA) demonstrated a considerable colocalization of PfCYP19B with PfBiP (**Fig 3B**). In the late ring stage, PfCYP19B/PfBiP fluorescence staining exhibits a punctuate pattern localized to an intracellular compartment in the vicinity of the nucleus that likely represents the early forms of the ER in the ring stages. In the later trophozoite stages, the presumed PfCYP19B/PfBiP ER-based colocalization signal expands into the parasite cytoplasm, while in schizonts, it fragments presumably following ER division into newly formed merozoites. Taken together, these results suggest that PfCYP19B, similarly to PfBiP, localizes predominantly in the ER for the most part of the IDC.

To test this further, we conducted a pull-down assay using *Pfcyp19b*-OE. Indeed, western blotting analysis revealed a direct interaction between the two proteins showing a considerable amount of PfBiP being pulled down using episomal HA-tagged PfCYP19B (**Fig 3C**). To explore this further, we searched for PfCYP19B putative interacting protein partners by coupling a native pull-down assay of HA-tagged PfCYP19B with mass spectrometry using 3D7 cell samples from three major IDC stages, rings (12 HPI), trophozoites (24 HPI), and schizonts (36 HPI). Confirming previous IFA and western blot results, the highest confidence interacting partner of PfCYP19B was PfBiP (**Table 1 and S1 Data)** [61]. In addition, PfCYP19B exhibits varying but overlapping interaction profiles across the three developmental stages, with most of its partners functionally relating to protein metabolism. These include protein disulfide-isomerase (PF3D7_0827900—PDI8; rings and trophozoites) and endoplasmin (PF3D7_1222300—GRP94; trophozoites), both of which were previously shown to interact with cyclophilin homologues in mammalian cell lines [62]. In addition, a thioredoxin-related protein (PF3D7_1352500) carrying a disulfide isomerase active site domain was pulled down with PfCYP19B in trophozoites. In the ring and trophozoite stages, PfCYP19B also interacts with proteins implicated in protein metabolism including several ribosomal subunits and ribosome processing proteins (PF3D7_1207100—ESF1; PF3D7_0729400—BRX1). Interestingly, in the ring stage, PfCYP19B interacts with several apical proteins (PF3D7_1200600—SHLP2, PF3D7_0503000 - 50S L28) some predicted to be involved in the invasion, as well as exported proteins (PF3D7_0831400) [61,63,64]. Although it is likely that these interactions occur during the trafficking process, currently we cannot rule out the possibility of PfCYP19B also binding the exported proteins in their final localization. In schizonts, PfCYP19B seems to interact with a smaller number of proteins; however, its strong association with PfBiP is retained which also agrees with the IFA colocalization study above (**Table 1 and Fig 3B**). Taken together, these results are compatible with the predicted role of PfCYP19B in the protein metabolism being principally associated with PfBiP but also with other factors. Like in most eukaryotic cells, PfBiP is expected to act as one of the key regulators in *P*. *falciparum* ISR [51,57,65] possibly linking the upregulation of PfCYP19B with PPQ and artemisinin resistance via these functionalities [35]. Moreover, at least one of the PfCYP19B interacting partners, thioredoxin-related protein (PF3D7_1352500), was also found to be differentially expressed in *P*. *falciparum* resistant parasites after artemisinin treatment *in vivo* in natural infections [31].

**Table 1:** Top mass-spectrometry hits from PfCYP19B pull-down experiment.

Accession	Description	Symbol	Coverage [%]	# Peptides	# PSMs	Function	Ratio CYP19B/EVC	present in # of replicates
Rings								
PF3D7_0917900 *	heat shock protein 70	BIP	44	26	95	protein folding, stress response	26	3
PF3D7_1115600 *	peptidyl-prolyl cis-trans isomerase	CYP19B	56	9	39	protein folding, stress response,	200	3
PF3D7_0821700 *	60S ribosomal protein L22	-	7	1	10	ribosome	6	3
PF3D7_1402500 *	ribosomal protein S27a	-	15	2	8	ribosome	7	3
PF3D7_1200600 *	shewanella-like protein phosphatase 2	SHLP2	18	5	7	cell invasion, apical complex	4	3
PF3D7_1026000	conserved Plasmodium protein	-	4	1	3	unknown	3	2
PF3D7_0831400	Plasmodium exported protein	-	4	1	2	exported	6	3
PF3D7_0729400	ribosome biogenesis protein BRX1	-	5	2	2	ribosome maturation	4	3
PF3D7_0827900	protein disulfide-isomerase	PDI8	2	1	2	protein folding, stress response	3	3
PF3D7_1368400	ribosomal protein L1	-	9	2	2	ribosome	3	3
PF3D7_0102500	erythrocyte binding antigen-181	EBA181	1	1	1	exported, cell adhesion, micronemes	5	1
PF3D7_0928500	conserved Plasmodium protein	-	13	1	1	unknown	4	2
PF3D7_1207100	pre-rRNA-processing protein ESF1	-	1	1	1	ribosome maturation	4	3
PF3D7_0503000	50S ribosomal protein L28	-	6	1	1	ribosome, apicoplast	4	3
PF3D7_0510500	topoisomerase I	TopoI	1	1	1	DNA metabolism	3	3
PF3D7_1136400	signal recognition particle subunit SRP72	SRP72	1	1	1	transport	3	3
Trophozoites								
PF3D7_0917900 *	heat shock protein 70	BIP	46	26	84	protein folding, stress response	30	3
PF3D7_1115600 *	peptidyl-prolyl cis-trans isomerase	CYP19B	56	9	34	protein folding, stress response,	200	3
PF3D7_1110400 *	RNA-binding protein	-	2	3	6	unknown	4	3
PF3D7_0814000 *	60S ribosomal protein L13-2	-	20	4	4	ribosome	4	3
PF3D7_1352500	thioredoxin-related protein	-	9	2	3	stress response	200	2
PF3D7_1020000	RNA-binding protein 34	RBM34	4	1	3	unknown	5	3
PF3D7_0210100	60S ribosomal protein L37ae	-	26	2	3	ribosome	5	3
PF3D7_0827900	protein disulfide-isomerase	PDI8	4	2	3	protein folding, stress response	4	3
PF3D7_0614500	60S ribosomal protein L19	RPL19	10	2	2	ribosome	5	3
PF3D7_0928500	conserved Plasmodium protein	-	13	1	2	unknown	4	3
PF3D7_1222300	endoplasmin	GRP94	1	1	1	folding, export	200	2
PF3D7_1207100	pre-rRNA-processing protein ESF1	-	3	1	1	ribosome maturation	200	3
PF3D7_1303800	conserved Plasmodium protein	-	0	1	1	unknown	5	2
PF3D7_0723900	RNA-binding protein	-	1	1	1	unknown	3	3
Schizonts								
PF3D7_0917900 *	heat shock protein 70	BIP	42	23	68	protein folding, stress response	23	3
PF3D7_1115600 *	peptidyl-prolyl cis-trans isomerase	CYP19B	43	8	31	protein folding, stress response	200	3
PF3D7_0614500	60S ribosomal protein L19	RPL19	5	1	2	ribosome	200	2
PF3D7_1033300	conserved protein	-	8	1	1	unknown	3	2

Number of detected peptides and peptide-spectrum matches (PSM) are shown. Column “Ratio CYP19B/EVC” indicates the ratio between signal abundance from pull-down performed on *Pfcyp19b*-OE parasite line over signal abundance in the empty Vector control (EVC, background). Only samples with a ratio ≥ 3 are shown in this table; ratios of 200 indicate that there was no peptide signal detected in the control background sample (*see* Materials and Methods). Pull-downs were performed independently in three biological replicates per each distinct IDC stage and mean values are shown.

*—High-confidence hits (present in all 3 out of 3 replicates

#PSM > 3, *Pfcyp19b*-OE/EV ratio ≥ 3).

### Transcriptional upregulation of *pfcyp19b* is mediated by short tandem repeat polymorphisms (STRs) in the gene’s promoter region

**Fig 4:**
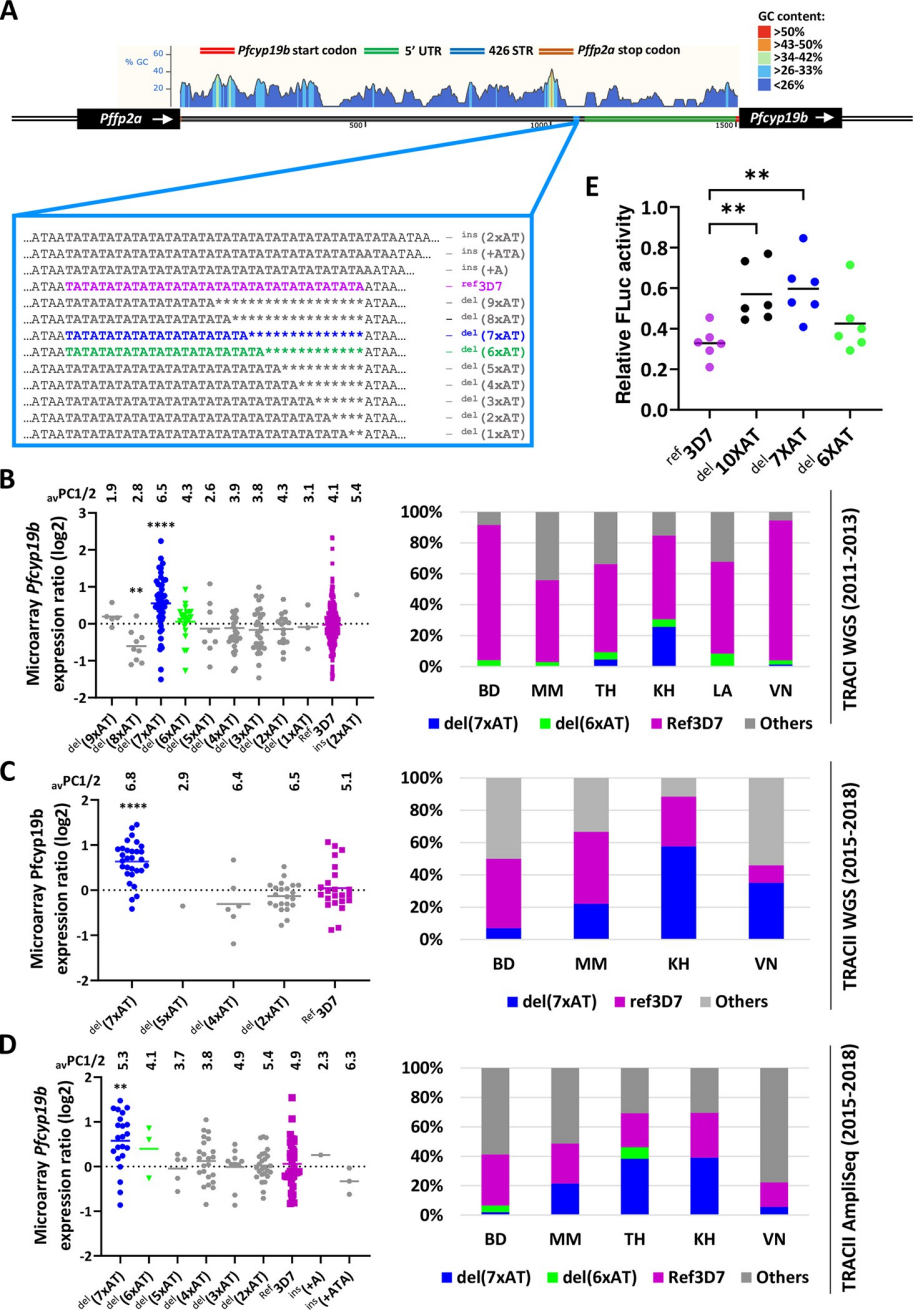
*Pfcyp19b* promoter region harbors short tandem repeat polymorphism that affects PfCYP19B expression levels. (A) Graphical representation of 1.5Kb-long intergenic region between falcipain 2a (FP2A) and *pfcyp19b* locus used in a dual luciferase reporter assay. Cyclophilin’s start codon is shown in red and the predicted 5’UTR in green. 426 polymorphic STR site has been highlighted in blue and has been magnified to show all distinct alleles detected in TRACI and TRACII genome analyses. The mountain chart (above) illustrates GC content (in % of GC) across the region. 426 STR is in the middle of AT-rich area just before the start of 5’UTR. (B-D) *Pfcyp19b* expression values according to detected 426 STR allele variants (dot plots, left panels) from TRACI WGS (n = 462—subfigure B) and TRACII (n = 87 for WGS—subfigure C; n = 144 for amplicon sequencing—subfigure D). Average parasite PC1/2 values for each STR variant are indicated above the dot plots. Charts on the right show geographic distribution (in %) of each STR variant in each GMS country. In TRACI ^del^(7xAT) variant was predominantly present in highly artemisinin-resistant Cambodian samples whereas ^ref^3D7 was a dominant allele in the whole GMS region. In TRACII ^del^(7xAT) variant spread across entire GMS region including still artemisinin-sensitive wGMS (Bangladesh, Myanmar). All *p*-values are derived from unpaired t-test with Welch’s correction performed individually between ^ref^3D7 and respective STR variants (E) Dual luciferase reporter assay of *pfcyp19b* promoter with genetically engineered STRs at position 426bp upstream of *pfcyp19b* start codon. Firefly luciferase signal has been normalized to Renilla luciferase used as a control for transfection and expression efficiency. All *p*-values are derived from unpaired t-test with Welch’s correction between ^ref^3D7 and respective STR variants where: * *p*<0.05, ** *p*<0.01, *** *p*<0.001, **** *p*<0.0001. All transient transfection dual luciferase experiments were performed in six independent biological replicates.

The main motivation of this study was the significant transcriptional upregulation of *pfcyp19b* observed in natural infections with *P*. *falciparum* in patients with delayed parasite clearance time after ACT treatment—the key phenotype of artemisinin resistance [31]. Here we showed that overexpression of PfCYP19B in *P*. *falciparum in vitro* indeed can drive limited but significant resistance in a stage-specific manner (**Fig 1D**). As such, PfCYP19B overexpression may provide a considerable parasite survival advantage within *P*. *falciparum* population in natural infections that are under strong drug selection pressure. As such, it is feasible to suggest that *pfcyp19b* upregulation is genetically underlined and being selected by the persistent application of the ART/PPQ ACT used in the GMS over the last decade [5]. Given that no copy number variations were reported for the *pfcyp19b* locus, we wished to search for the putative causative genetic variation(s) responsible for *pfcyp19b* upregulation. We carried out amplicon sequencing (AmpliSeq) of the locus comprising *pfcyp19b*, and its neighboring genes encoding falcipain 2a&b and falcipain 3, AP2 domain transcription factor, and PF3D7_1115800 conserved Plasmodium protein (18.7 Kb region surrounding p*fcyp19b* locus on chromosome 11, pos. 578385 to 597119) **(S9 Fig)**. By this approach, we determined a high-fidelity sequence of the *pfcyp19b* locus for 144 samples of the TRACII cohort involving parasites with variable degree of artemisinin resistance. Crucially, we detected a polymorphic locus (labeled as “426 STR”) just 426bp upstream (at position 591054) of *pfcyp19b* start codon (**Figs 4A and S9 and S2 Data)**. This consisted of multiple alleles of a sequence length polymorphism at a short tandem repeat (STR) in a highly AT-rich region, in the near vicinity of the bioinformatically predicted 5’UTR region of the *pfcyp19b* predicted gene model significantly associating with *pfcyp19b* expression (eSTR) (*p* = 1.60E-04) (**S2 Data**) [66]. The predicted transcription start site (TSS) is located only 16 bp downstream of this STR [66]. In this set of 144 TRACII samples, we have detected 9 distinct alleles with polymorphisms of AT-dinucleotide stretches ranging from nine deletions of AT to up to two AT insertions as compared to the locus in the reference genome sequence of the *P*. *falciparum* 3D7 strain (^ref^3D7) (**Fig 4A and S2 Data**). Additionally, we detected two insertions of non-pair character including a single A base and ATA triplet (**Fig 4A and S2 Data**).

Guided by this result, we inspected the same locus extracted from whole genome sequencing (WGS) datasets from TRACI and TRACII *P*. *falciparum* samples aiming to assess the geographical and temporal occurrence and frequency of these sequence length polymorphisms throughout the GMS parasite population. Depth of the locus sequence coverage for parasites from TRACI (mean coverage = 71.7) and TRACII (mean coverage = 108.9) cohorts provided sufficient information to allow confident estimation of the *pfcyp19b* SRT length polymorphisms allele *in vivo* **(Table 2)**. Overall, we detected 13 alleles in the GMS *P*. *falciparum* samples in the combined TRACI and TRACII datasets using WGS and AmpliSeq methodology **(S2 Data)**. Interestingly, in TRACI there was a skewed geographical distribution of ^del^(7xAT) allele in the regions with a high occurrence of artemisinin resistance such as Cambodia (KH), Vietnam (VN), and Thai-Myanmar (TH) border (**Fig 4B–4D, right bar graphs**). ^del^(7xAT) was progressively increasing in frequency from TRACI (2011–2013) to TRACII (2015–2018) and steadily substituting the initially dominant reference 3D7 (^ref^3D7) allele. While in TRACI the ^del^(7xAT) was only found sporadically in KH and TH sites, in TRACII this allele spread to all subregions to some degree including wGMS regions of Bangladesh (BD) and Myanmar (MM) **(Fig 4B–4D, right bar graphs).** Nevertheless, the sites with low occurrence of drug resistance such as Bangladesh and Vietnam in 2011–2013 (TRACI) and Bangladesh in 2016–2018 (TRACII) were largely characterized by high frequencies of the wild type allele and/or the “intermediate” remaining alleles ^del^(2x-6xAT).

Importantly there was a strong statistically significant association of ^del^(7xAT) and *pfcyp19b* upregulation (*p* = 3.51E-06) when compared with parasites carrying the wild type (^ref^3D7) allele in the TRACI sample set (**Fig 4B, left scatter plots**). This association between ^del^(7xAT), and *pfcyp19b* overexpression was reproduced in the TRACII study (*p* = 7.13E-05 for WGS, *p* = 2.57E-03 for AmpliSeq) and was also supported by linear correlation in AmpliSeq dataset between STR deletion length and average *pfcyp19b* expression level (PCC = 0.69, insertions have been excluded from the analysis due to very low number of samples) (**Fig 4C and 4D, left scatter plots**). Higher levels of *pfcyp19b* expression also linearly correlated with higher PC1/2 of the parasites in TRACI study (PCC = 0.64). Accordingly, the ^del^(7xAT) deletion segregates with increased PC1/2 while the wild type allele is present mostly in fast-clearing parasites found in the TRACI study **(Fig 4B)**, but not in TRACII (**Fig 4C and 4D**). We have not detected ^del^(8xAT) and ^del^(9xAT) alleles in the TRACII study. Based on these results we speculate that the remaining shorter alleles represent intermediate steps towards optimal deletions, such as ^del^(7xAT), that in the end is driving transcriptional upregulation and as such contributing to artemisinin resistance.

To investigate whether the detected STR polymorphism can alter the transcriptional activity of the *pfcyp19b* promoter, we carried out *in vitro* transient transfection assays with luciferase reporter genes as previously described [67, 68]. For that, the ~1.5Kb region, upstream of *pfcyp19b* start codon including the promoter with specifically engineered STR lengths (^ref^3D7, ^del^(10xAT), ^del^(7xAT), ^del^(6xAT)) were cloned upstream of the luciferase gene in the transient transfection vector (**Fig 4A,** *see* Materials and Methods). Indeed, the promoter with ^del^(7xAT) deletion exhibited 82% higher expression levels when compared to wild type allele ^ref^3D7 (*p* = 5.11E-03) (**Fig 4E**). Additionally, ^del^(10xAT) (an artificially created STR, not detected in natural infections) also exhibited higher expression levels when compared to ^ref^3D7 (*p* = 7.16E-03). Interestingly, the ^del^(6xAT) failed to yield higher expression compared to the 3D7 reference (corroborating *in vivo* observations) suggesting that the length of the STR polymorphism needs to be very subtle and specific to exert phenotypic difference (**Fig 4E**).

**Table 2:** Coverage of examined *pfcyp19b* locus in TRACI and TRACII studies.

Study	TRACII AmpliSeq	TRACI genome	TRACII genome
**No of samples**	144	461	365
**Target mean coverage**	2731.88	71.71	108.88
**Target median coverage**	2784.98	65.89	116.17
**Coverage SD**	644.42	28.52	62.27
**Range of coverage**	45.6–4127.9	6.9–213.5	0.3–375.1

Average coverage for 18.7 Kb long *pfcyp19b* amplicon and the coverage of the same fragment extracted from TRACI WGS and TRACII WGS data. Numbers indicate number of reads on average covering the analyzed locus in each respective dataset.

## Discussion

### PfCYP19B function within the ISR pathway contributes to drug resistance

Here, we showed that overexpression of PfCYP19B gives *P*. *falciparum* parasites limited but significant resistance/resilience to two key antimalarial drugs, namely DHA (and presumably other artemisinin derivatives) and PPQ (**Fig 1D**). This is likely mediated by the PfCYP19B peptidyl-prolyl isomerase activity in protein folding, taking part in the ISR while countering the damage of proteins and other intracellular structures exerted by both drugs. This is analogous to other eukaryotic systems where PfCYP19B orthologues were implicated in proteotoxic and oxidative damage responses [53,62]. Overexpression of a mammalian orthologue of PfCYP19B in rats has been previously shown to suppress apoptosis induced by ER stress resulting from prolonged accumulation of misfolded proteins and its inhibition rendered cells more vulnerable to ER stress [62]. Accordingly, the depletion of cyclophilin B leads to the dysregulation of redox homeostasis in the endoplasmic reticulum [69]. Indeed, artemisinin-derivates are known to cause misfolded protein aggregation, severe ER stresses, and UPR induction, all of which is a response to the drug-induced proteotoxic shock [35,47]. For PPQ, a recent study showed that knockout of G protein-coupled receptor (GPCR)-like *PfSR25* in *P*. *falciparum* caused the parasites to become more susceptible to oxidative stress and PPQ, supporting the idea that PPQ MOA might be related to oxidative stress and/or proteotoxicity [70]. Hence, upregulation of PfCYP19B in *P*. *falciparum* parasites has a direct potential to alleviate drug-induced proteotoxic damage giving the parasite survival advantage at least to some degree.

Indeed, exposure of *P*. *falciparum* parasites to both DHA and PPQ seems to affect the levels of eIF2α-P, that in most eukaryotic cells represents the central signaling protein of the ISR [51]. eIF2α-P mediates induction of the ISR that allows the eukaryotic cells to recover their homeostatic balance after stress factors such as heat shock, haem deprivation, starvation, excessive oxidation, or accumulation of misfolded proteins [51]. eIF2α-P was also shown to play a role in the stress response in *P*. *falciparum* in which elevation of PfeIF2α-P causes developmental arrest and suppression of proteosynthesis [48,71]. The effect of artemisinin on the levels of PfeIF2α-P shown in this study (**Fig 2**) was demonstrated previously [48]. Crucially, here we showed that overexpression of PfCYP19B can suppress the DHA-induced eIF2α-P to significantly lower levels. Such suppression could reduce the parasites’ sensing of the drug-stimulated proteotoxic shock and hence, lessening the effect of presumed downstream apoptosis-like events. This model is consistent with a previous study, showing that artemisinin-resistant parasites can reduce the levels of artemisinin activation in the cells, leading to reduced proteotoxicity and increased survival rate [33].

Somewhat surprisingly, PPQ appeared to also affect eIF2α-P although in the opposite direction. This is quite unexpected given the PPQ MOA and resistance mechanism was mostly linked with hemoglobin degradation in the digestive vacuole (DV), similar to other quinolines [54,72]. However, there is previous evidence showing antagonistic interactions between PPQ and DHA in several P. *falciparum* cell lines [30]. Here we showed that PPQ MOA is linked with CsA cytotoxic activity in an antagonistic manner that can be modulated by the levels of PfCYP19B (**Fig 1E**). Hence, it is feasible to speculate that similar to the artemisinins, PPQ MOA also involves some form of proteotoxicity/oxidative damage inducing ISR and that overexpression of PfCYP19B has the potential to contribute to *P*. *falciparum* resistance/resilience mechanisms to both drugs (simultaneously). In both cases, PfCYP19B acts as a negative regulator of ISR sensing (via PfeIF2α-P) and thus, possibly allowing parasites to progress through the IDC avoiding presumed apoptosis-like events induced by DHA and PPQ. Indeed, in two extensive studies by Zhang et al., the authors found that Dd2 parasites harboring C580Y mutation have surprisingly higher basal (no drug treatment) levels of phosphorylated PfeIF2α when compared to the wild type strain [47,48]. This suggests that mutant parasites reduce global protein translation and are under some form of constant ER stress. Interestingly, upon DHA treatment (90 min, 500 nM) PfeIF2α-P levels in *pfk13* mutants remained almost unchanged. Contrarily, the levels of PfeIF2α-P in wild type parasites increased significantly [47]. Additionally, inhibition of PK4 kinase (PfeIF2α-specific kinase) reduced artemisinin resistance and abolished recrudescence. Taken together these results are consistent with a model in which PPQ treatment somewhat “phenocopies” the effect of *pfk13* SNPs on PfeIF2α-P and acts opposite to DHA upon cellular stresses such as artemisinin treatment.

### Artemisinin/multidrug resistance of malaria parasites is a complex genetic trait

Although the nonsynonymous SNPs within the PfK13 KELCH domain are currently the most robust markers, it is becoming progressively clear that artemisinin resistance is a complex genetic trait that consists of a multitude of genetic alterations [73]. These alterations presumably create a suitable physiological state of the malaria parasite that enables its survival under physiologically relevant drug exposures in PfK13-dependent but also possibly in independent manners [14]. Currently little is known regarding the mechanisms of such a genetic background, however, several population transcriptomic analyses suggested that at least part of it could be mediated by altered expression levels of broad but defined sets of genes [5,31,35]. Recently, we identified the Artemisinin Resistance-associated Transcriptional Profile (ARTP) consisting of 156 genes, most of which relate to biological functions that have high potential to contribute to artemisinin resistance as shown by numerous *in vitro* studies [32–34,74–76]. The ARTP correlates particularly well with the results of two recent system biology explorations of artemisinin resistance using the PiggyBac transposon mutagenesis [77] and omics studies of *pfk13* SNP transgenic cell lines [37]. In both studies, artemisinin resistance was shown to be predominantly driven by alterations in pathways such as protein folding, ER stress, and various components of the ISR pathway. At least four independent *in vitro* studies reported PfCYP19B to be upregulated either at the transcript or the protein level. In three independently generated *pfk13* mutants (R539T, N458Y, and C580Y) authors found a marked increase in *pfcyp19b* transcripts [78,79]. Most recently, two mutants (Cam3.II R539T and Cam3.II C580Y) were shown to have increased levels of PfCYP19B peptides by comparative mass spectrometry analysis (**S2 Fig**) [37]. Additionally, artemisinin-resistant 3D7-11R lines generated by long-term artemisinin treatment and not carrying any of *pfk13* nonsynonymous mutations were also found to have increased transcript levels of *pfcyp19b* [40]. Those two large-scale explorations, along with several above-mentioned *in vitro* studies, support the role of PfCYP19B in artemisinin resistance directly or indirectly. In this model, upregulation of PfCYP19B can drive a limited but significant level of resistance by itself (shown here) but will likely contribute to a much stronger effect when combined with other genetic variations of the multifaceted artemisinin resistance mechanism [73]. Moreover, like PfK13, PfCYP19B can co-function with several existing exonic mutations associated with artemisinin resistance in the GMS as well as with those newly emerging in Africa. These include mutations in the main hemoglobinase enzymes such as falcipain 2a/b and 3 whose genes are nearest genomic neighbors of *pfcyp19b* (**S9 Fig**). Falcipain genes (or their genetic alterations) were shown to drive artemisinin resistance *in vitro* using both forward and reverse genetic studies [40,80]. Moreover, their exonic mutation(s) and altered expression were also linked with artemisinin resistance in clinical isolates [31,81]. It is feasible to speculate that the PfCYP19B upregulation can be co-selected with the genetic alterations (both SNPs and/or transcript upregulation) of the falcipain genes in expression quantitative trait loci (eQTL)-like manner giving it a stronger selective advantage. In future studies it will be interesting to investigate *pfcyp19b* eQTL linkages with falcipain but also other genes such as coronin, that are currently emerging as new markers of artemisinin resistance [81].

### STR sequence length polymorphisms modulate transcriptional regulation

Addressing possible genetic bases of PfCYP19B upregulation, we identified a sequence length polymorphism at a short tandem repeat (STR) sequence element in its promoter region. There was a statistically significant association with the ^del^(7xAT) deletion and *pfcyp19b* transcriptional upregulation *in vivo* (**Fig 4B–4D**), and *in vitro* demonstrated by the dual luciferase reporter assay (**Fig 4E**). Indeed, AT-rich STRs are known to account for more than 10% of the entire *P*. *falciparum* genome with a particular overrepresentation in the regions flanking the gene’s transcriptional start sites (TSS) [82]. Moreover, insertion/deletion polymorphisms (Indel) at the STR sequence were shown to account for a considerable fraction of *P*. *falciparum* polymorphisms in *in vitro* cultured parasites, derived from genetic crosses [83]. Exploring the reference MalariaGEN global *P*. *falciparum* dataset, Han *et al*. recently demonstrated that STR polymorphisms can differentiate distinct global parasite populations analogously to exonic SNPs suggesting their dynamic evolution [84]. Here, we are the first to demonstrate an association between STR sequence polymorphisms and transcriptional regulation with possibly direct implications on transcriptional regulation in malaria parasites, and, as a consequence of increased *pfcyp19b* expression, on a crucially important drug resistance phenotype. This is consistent with the emerging evidence in higher eukaryotes, including human, where STR sequence polymorphisms are being progressively implicated in a broad array of genetic traits as effectors of transcriptional variations [85,86].

Taken together, our observations provide a plausible hypothesis that highly polymorphic STR sequences in the promoter regions of *P*. *falciparum* genes can modulate their expression, giving the parasite the potential for a highly dynamic fine-tuning of transcript levels of individual genes. Given the high fluidity of the AT-rich P. falciparum genome, many of the STR sequence polymorphisms arise mitotically in a microsatellite-like fashion during DNA replication of asexual growth stages via, likely polymerase slippage mechanism. This could create highly dynamic transcriptional variability giving parasite potent ways to respond to selection pressures. In light of these findings, future studies investigating the full extent of the STR-driven transcriptional variability and its interaction with exonic polymorphisms for the parasite’s phenotypic plasticity, including drug resistance are warranted.

## Materials and methods

### Ethics statement

All samples used for this study were collected with written informed consent from the patients or their legal guardians. All protocols were approved by the Oxford Tropical Research Ethics Committee.

### Parasites used in the study

*P*. *falciparum* TRACI and TRACII clinical isolates collected at patient’s admission (hour 0) (RNA-Seq and WGS): Figs 1A, 1B and 4B–4D, and S1.

*P*. *falciparum* 3D7 laboratory strain: Figs 1C–1E, 2A–2E, 3A–3C and 4E, S1, and S4–S8 and Table 1.

*P*. *falciparum* Dd2 laboratory strain: S2 Fig.

*P*. *falciparum* Cam3.II isogenic Cambodian strain: S2 Fig.

### *In vivo* sample collection

All samples used in this study were collected from patients involved in Tracking Resistance to Artemisinin Collaboration (TRAC) I and II, a multi-site clinical trials that took place between 2011–2013 (TRACI) and 2015–2018 (TRACII). All details regarding samples collection, site locations, inclusion criteria, parasitemia assessment, and given treatments were published previously [1,3].

### *In vivo* transcriptome and genome sample datasets

All transcriptomes analyzed in this study were derived from previously published data available on GEO depository (TRACI Microarray: GSE59099; TRACII Microarray: GSE149735; TRACII RNASeq: GSE169520) [35,87]. All RNA expression levels are representative of the parasite transcriptome state at the time of blood collection at the admission to the hospital for the treatment–“hour 0”.

All genomes analyzed in this study were derived from studies previously published [88,89]. This publication uses data generated by the Wellcome Trust Sanger Institute as part of the MalariaGEN Plasmodium falciparum Community Project and the Pf3k Project, as described in Miotto, Amato et al, Nature Genetics, 2015 (doi:10.1038/ng.3189) [88] and Jacob CG, Thuy-Nhien N, Mayxay M, et al, eLife, 2021 (doi:10.7554/eLife.62997) [89].

### gDNA extraction

All samples used for genomic DNA extraction required for the study of *in vivo* STR polymorphism of *pfcyp19b* promoter region were derived from TRACII clinical trials samples collected at patients’ admission. gDNA used for TRAC2 amplicon sequencing was extracted from TRIzol (Invitrogen) fractions after the removal of total RNA. In brief, Buffer containing 4M guanidine thiocyanate (Sigma), 50mM sodium citrate (Sigma) and 1M Tris (BioRad) was mixed with organic TRIzol fraction (bottom phenol phase) and incubated at room temperature for 10 minutes with continuous agitation and subsequently centrifuged at 10000 x g. The aqueous phase containing solubilized genomic DNA was aliquoted from the top fraction and treated with RNAse A (Qiagen) and Proteinase K (Qiagen). 100% ethanol was added, and the mixture was subsequently transferred onto QIAamp DNA Blood column (Qiagen) and gDNA was extracted following manufacturer’s protocol. Eluted genomic DNA was vacuum desiccated at room temperature and resuspended in nuclease-free water. DNA samples were quantified with Qubit DNA High Sensitivity kit (Invitrogen) and stored at -20°C.

gDNA used for *in vitro* transfection validation and qPCR analysis was extracted using Quick-DNA kit (ZYMO) following manufacturers instruction.

### *In vitro* culture

Plasmodium falciparum 3D7 MR4 strain (wild type) was maintained in purified human pRBC in RPMI 1640 medium (Gibco) supplemented with Albumax I (Gibco) (0.25%), hypoxanthine (Sigma) (0.1 mM), Sodium bicarbonate (Sigma) (2 g/L), and gentamicin (Gibco) (50 μg/L). Cultures were kept at 37°C with 5% CO_2_, 3% O_2_, and 92% N_2_. Culture media were replenished every 24 hours. Freshly washed pRBC (Interstate Blood Bank) were added to the culture when necessary. Both parasitemia and parasite morphology were assessed by microscopic examination of blood smears stained with Giemsa (Sigma).

### Parasite synchronization

For tight synchronization of parasites, we employed a combination of Percoll and sorbitol synchronization method as described [38]. In brief, parasite cultures were routinely synchronized with 5% (w/v) sorbitol for several cycles before the assay to keep tight developmental window for schizont enrichment. At around 5–10% parasitemia mature segmenting schizonts, were enriched using 75% Percoll (Sigma) solution with 15min centrifugation at 1000g. The layer of segmented schizont was collected and washed once with media. Freshly washed pRBCs were added to 10% hematocrit and cells were incubated for precisely 3 hours with agitation to achieve single invasions in red blood cells. After 3 hours parasite culture was synchronized with 5% (w/v) sorbitol and adjusted to desired parasitemia and hematocrit for further assays.

### Plasmid construct generation and episomal overexpression system

Peptidyl-prolyl cis-trans isomerase overexpression system (*Pfcyp19b-*OE) was designed as described previously [40]. In brief, full length *pfcyp19b* (PF3D7_1156000) cDNA was derived from *P*. *falciparum* 3D7 strain with primers 5`-ATCGGGATCCATGAATAAATTAGTGTCAATCATT-3`and 5`- ATCGGCTAGCCAATGGCAATTCTCCTGATTC-3`. NheI restriction site on the 5’ end of the forward primer and the BamHI restriction site on the 5’ end of the reverse primer were added to allow for unidirectional cloning into the multiple cloning site (MCS). Fragments were subsequently inserted into pBcamR_3xHA vector at the BamHI/NheI sites on the MCS upstream of the triple hemagglutinin (HA) sequence in order to obtain an HA-tagged protein product. Plasmid was then transfected into 3D7 parasite strain and maintained by positive selection with blasticidin (BSD, Sigma) (10μg/mL initially and then 5μg/mL, no BSD during drug treatment assays). A control parasite line (empty Vector control) was additionally generated by transfecting 3D7 parasites with an empty pBcamR_3xHA plasmid and grown in an equal concentration of BSD alongside the overexpression parasite line. To generate plasmid for GFP/FKBP-tagged PfCYP19B, PfCYP19B open reading frame was PCR amplified from *P*. *falciparum* 3D7 genomic DNA with primers 5`- TAAGCAGCGGCCGCTAATGTGTTTATATATATA-3`and 5`- TGCTTACCTAGGCAATGGCAATTCTCCTGA-3`and cloned into the NotI/AvrII sites of pSLI-Sandwich in-frame with the coding sequences of 2xFKBP-GFP-2xFKBP-NeoR. The first attempt at generating a knockout was done using targeted gene disruption with selection-linked integration as described before [90]. The truncated gene would contain the first 129 amino acids, followed by C-terminal tagging with GFP, a skip peptide, and a drug selection marker. The homology regions correspond to the region cloned into the plasmid using primers 5`- TGCTTAACGCGTCATTGATAATAATCCTCTTTTAC-3`and 5`-TAAGCAGCGGCCGCTAACTATCGATGACAAGCCAC-3`. These were cloned into the NotI/MluI site. Episomal selection of parasites transfected with this plasmid failed, indicating that parasite uptake of the plasmid was unsuccessful. For transfection, ring-stage parasites were transfected with 50 μg of purified plasmid DNA (Qiagen) using the GenePulser (Bio-Rad) as previously described [90]. Selection of transfectant lines was done with 4 nM WR99210 and 0.4 mg/mL G418 (for GFP/FKBP-tagged strain; Sigma). Plasmid maps representing each plasmid mentioned above are depicted in **S10 Fig**.

### *In vitro* drug sensitivity assays

Parasite lines episomally overexpressing PfCYP19Bx3HA (*Pfcyp19b-*OE) and empty Vector control were synchronized as described above. Highly synchronous parasites at 4 HPI, 20 HPI and 35 HPI were then dispensed to 96 well plates containing 12 serially diluted concentrations of DHA or PPQ (Sigma-Aldrich) to make final 1% hematocrit and 1% parasitemia. The top concentration of compounds used in was 1,000 nM for PPQ and 200 nM for DHA followed by 2-fold serial dilution and a drug-free control. 4 hpi, 20 hpi, and 35 hpi parasites were incubated with the drug for 72, 56 and 41 hours respectively to allow all parasites to invade and proceed to the next cycle. For *in vitro* drug sensitivity assay with or without 80 nM CsA supplementation (Fig 1D), mid ring stage (approx. 12 HPI) of *Pfcyp19b-*OE and Vector control strains were used. The top concentration of compounds used was 1,000 nM for PPQ, MFQ, AQ, CQ, and PYR, 100 nM for DHA, and 20 nM for ATQ. The number of new, viable parasites in each well on the subsequent replication cycle was quantified by flow cytometry. Cells were stained using 50 μL of 8 μM Hoechst 33342 in PBS (pH 7.2) for 15 minutes at 37°C, followed by addition of ice-cold 200 μL of PBS. Cells were quantified using LSR Fortessa X-20 Flow Cytometer (BD Biosciences) using UV laser (355nm) and results were analyzed with FACS Diva Software (BD Biosciences). Dose-response curves and IC_50_ estimation were obtained using GraphPad Prism 8 four-parameter dose-response curve equation (GraphPad Software Inc.). All assays were performed in biological triplicates and additionally in technical duplicates per dose. Corresponding strain with an empty vector at the same generation cycle was used parallelly as a control. Two-tailed heteroscedastic t-test (paired/unpaired as indicated) was used to calculate significance.

### Ring survival assay

Ring survival assays (RSA) were performed based on previously published protocol [38]. In short, double synchronized early ring stage (0–3 hpi) parasites (see above) with 1% parasitemia and 2% hematocrit culture were incubated in a 24-well plate with 700 nM Dihydroartemisinin (DHA). Dimethyl sulfoxide (DMSO) (Sigma) was used as a control. Upon 6 hours of treatment, both DMSO and DHA treated parasites were washed three times with complete RPMI media and cultured for another 72 hours. Parasites at the end of the treatment were double stained with Hoechst 33342 (Thermo Fisher) and MitoTracker deep red FM (Thermo Fisher) and quantified for viable parasites using flow cytometry and microscopy. RSA result is valid when the control parasite growth rate (DMSO treated parasitemia after experiment / DMSO treated parasitemia before experiment) is higher than 1.5. Percentage survival (%) = DHA treated parasitemia / control parasitemia x 100%.

### Isobologram assays

Cytotoxic drug complementarity assessment drug assays were performed as described before [91]. In brief 200 μL of parasite culture (rings approx. 12HPI, 1% hematocrit and 1% parasitemia) of *Pfcyp19b*-OE and Vector control parasite strains were used. Transfectants were grown under 5 μM BSD pressure which was removed during the cycle when isobolograms were performed. Prior to the beginning of an assay, parasites were synchronized as described above. The top concentration of compounds used was 500 nM for PPQ, 4 μM for CsA and 500 nM for DHA followed by ten 2-fold serial dilutions and a drug-free control (DMSO). IC_50_ measurements were conducted for each drug alone and for drug combinations at fixed volumetric ratios (Drug A: Drug B = 5:0, 4:1, 3:2, 2:3, 1:4, 0:5). A total of 72 hours incubation time was done for all assays. Following incubation, supernatant was removed and iRBCs were stained with 50 μL of 8 μM Hoechst 33342 in PBS (pH 7.2) for 15 minutes at 37°C, followed by addition of ice-cold 200 μL of PBS. Cells were quantified using LSR Fortessa X-20 Flow Cytometer (BD Biosciences) using UV laser (355nm) and results were analyzed with FACS Diva Software (BD Biosciences). Raw FACS data was gated and analyzed with FACS DIVA software. Linear regression analysis of dose-response curves and IC_50_ determination was subsequently performed with GraphPad Prism. The isobologram analysis was performed according to the Loewe additivity model [92], where the sum of fractional inhibitory concentrations (ΣFIC50) for the two inhibitors in a combination is indicative of additivity, synergism or antagonism. The combinations with ΣFIC50>1 are considered antagonistic, <1 synergistic and if equal to 1 additive. ΣFIC50were calculated using the following equation: *Sum FIC* (Σ*FIC*) = *IC*50 *of A in mixture* / *IC*50 *of A alone* + *IC*50 *of B in mixture* / *IC*50 *of B alone*.

### eIF2α phosphorylation assays

Synchronized ring stage parasites (see above) (3D7 MR4, *Pfcyp19b*-OE and Vector control) of approximately 10HPI (10% parasitemia) were treated for 90 minutes with different antimalarial drugs–DHA (500 nM) (Sigma), PPQ (500 nM) (Sigma), CSA (4 μM) (Sigma), AQ (500 nM) (BEI Resources), PYR (800 nM) (BEI Resources), CQ (1 μM) (BEI Resources), ATQ (2 μM) (BEI Resources), MFQ (2 mM) (BEI Resources). Concentrations have been selected to fit within physiological concentration brackets. DMSO has been used as a solvent control. Co-treatments were performed with additional 500 nM PPQ presence. After treatment parasites were immediately liberated from RBCs with 0.1% saponin and total protein extracted (see below). Proteins were quantified using Pierce BCA Protein Assay Kit (Thermo Scientific). Samples were normalized and identical protein amount in identical volumes were loaded onto a gel and processed as described below. After blocking membranes were incubated for 4 hours (room temperature) with primary antibody (rabbit) (1:500, in 5% (w/v) BSA (Sigma) dissolved in 1% TBS-T (Sigma)) recognizing Plasmodium eIF2α phosphorylation at Ser51 (Cell Signaling) as published previously [34]. PfBiP rabbit antibody (1:10000) (and β-actin mouse antibody at 1:2000 (Sigma)) were used as loading controls. HA-tagged PfCYP19B protein was detected using rat anti-HA antibody (1:1000) (Roche). Primary antibodies were probed with secondary highly cross-absorbed anti-rabbit Alexa Fluor 488 (1:2000) (Jackson Antibodies), and highly cross-absorbed anti-rat Alexa Fluor 594 (1:4000) (Thermo Fisher). Membranes were washed thoroughly with 1% TBS-T and 1xPBS and visualized on ChemiDoc MP (BioRad). Fluorescence signal was analyzed using ImageJ (NIH). Quantification reflects relative amounts of each protein band normalized to the lane’s loading control (PfBiP) for which inverted pixel densities (256 grayscale) for all bands have been extracted and background subtracted. Complete uncropped images of the western blot membranes can be found in **S1 Images.**

### Protein extraction

For the stage-specific pull-down analysis parasites were harvested at ring stage (approx. 12HPI), trophozoites (approx. 24HPI) and schizonts (approx. 36HPI) Parasites were liberated, and red blood cells lysed using 10 volumes of 0.1% (w/v) saponin (Sigma) in ice-cold 1 x PBS (1^st^ Base) for 5 minutes. Parasite pellet was washed three times with 1 x PBS and the pellet was lysed (for most of assays except Pull-downs) with RIPA buffer (150 mM sodium chloride (Merck); 1% Triton X-100 (BioRad); 0.5% sodium deoxycholate (Sigma); 0.1% sodium dodecyl sulfate (BioRad); and 50mM Tris (BioRad) pH8.0) supplemented with 1% (v/v) protease inhibitor cocktail (Nacalai Tesque) and 1% (v/v) phosphatase inhibitor cocktail (Thermo Fisher). Samples were incubated for 30 min at 4°C with gentle agitation. For native pull-down assays RIPA buffer was substituted with Pierce IP Lysis Buffer (Thermo Fisher) with 1% (v/v) protease inhibitor cocktail (Nacalai Tesque) and 1% (v/v) phosphatase inhibitor cocktail (Thermo Fisher). Protein lysate debris was removed by centrifugation (15 min at 4°C) and protein concentration in the supernatant was quantified using Pierce BCA Protein Assay Kit (Thermo Scientific). Samples were frozen with liquid nitrogen and stored at -80°C.

### Western blot analysis

Western blots were performed following Abcam protocols (available on manufacturer’s website) with some modifications. The parasitized RBC fraction was initially lysed with ice-cold saponin (Sigma) (0.1%) in PBS (1st Base) (1x) and washed three times with ice-cold 1 x PBS. Free parasite pellet was lysed in RIPA buffer supplemented 1:100 (v/v) with EDTA-free protease inhibitor (Nacalai Tesque) and 1% (v/v) phosphatase inhibitor cocktail (Thermo Fisher). The lysate was spun, and protein concertation was measured using Pierce BCA Protein Assay Kit (Thermo Scientific). An identical amount of total protein (10–20 μg, depending on the assay) in RIPA or Pierce IP buffer was mixed with Fluorescence Compatible Sample Buffer (4X) (Thermo Scientific) supplemented with 2-mercaptoethanol (10%) (Sigma) and boiled at 100°C for 5 minutes. Identical amounts in identical volumes of protein lysates were separated using premade Mini-PROTEAN TGX Precast 12% native SDS-PAGE gel (Bio-Rad). Proteins were transferred on the nitrocellulose membrane (BioRad) using Trans-blot Turbo Transfer System using manufacturer’s original reagents (BioRad) 3 minutes for each gel at 2.5A constant current. Membranes were blocked with 5% (w/v) solution of Bovine Serum Albumin (BSA) (Sigma). For CYP19Bx3HA episomal expression validation HA-tagged protein was detected using 1:1000 rat anti-HA antibody (Roche) and 1:5000 goat anti-rat (HRP)-conjugated antibody (Abcam). Actin was used as a loading control and probed with 1:2000 anti-β-actin mouse monoclonal antibody (Sigma) and then subsequently with 1:5000 goat anti-mouse (HRP)-conjugated antibody (Abcam). For total PfCYP19B detection 1:2000 rabbit polyclonal anti-PfCYP19B (Genscript) antibody was used with 1:5000 rabbit anti-PfBiP (BEI Resources) as a loading control. Immunocruz Western Blotting Luminol Reagent (Santa Cruz Biotechnology) was used as a substrate for HRP, and the resulting chemiluminescence signal was acquired using Luminescent Image Analyzer LAS4000 System (Fujifilm) or ChemiDoc MP (for fluorescent antibodies) (BioRad) and analyzed using ImageJ (NIH). Complete uncropped images of western blot membranes can be found in **S1 Images.**

### Life cell immunofluorescence imaging

For live cell imaging, infected RBCs were washed with 1 x PBS (1^st^ Base) and stained at room temperature for 10 min with 4 nM Hoechst33342 (Sigma-Aldrich). Stained cells were then mixed with a droplet of 1 x PBS on a glass slide. Images were taken with a Zeiss Axio Observer Z1 equipped with Axiocam 503, viewed with 100x/1.40 Plan-Apochromat Oil DIC M27 objective lens. Images were overlaid and processed in Zen Blue (Zeiss).

### Fixed tissue indirect immunofluorescence assays (IFA)

Thin smears made from the synchronous culture of *Pfcyp19b*-OE lines were fixed using paraformaldehyde (Sigma) (4%) and glutaraldehyde (Sigma) (0.0075%) in 1xPBS (1^st^ Base) for 15 min, permeabilized in Triton X-100 (Sigma) (0.1%) for 10 min and then blocked with bovine serum albumin (BSA, Sigma) (3%) with glycine supplementation (1st Base) (22.52mg/ml) in PBS-T for 1 hour. Subsequently they were incubated with 1:200 rat monoclonal anti-HA antibody (Roche) and 1:200 rabbit polyclonal anti-PfBiP (BEI Resources) followed by incubation for 30 minutes with 1:2000 Alexa Fluor 594 goat highly cross-absorbed anti-rat (Jackson Immunoresearch) and 1:2000 Alexa Fluor 488 goat highly cross-absorbed anti-rabbit secondary antibodies (Jackson Immunoresearch). Nuclear counterstain was done with DAPI (Thermo Scientific) (1 ng/μl) for 10 min. All steps were performed at room temperature. Slides were mounted with ProLong Gold Antifade (Thermo Scientific). Images were captured with Zeiss LSM710 confocal microscope equipped with an Airyscan detector using a Plan-Apochromat 100x/1.46 oil objective and processed using Zen (Blue edition) imaging software (Zeiss). Images were analyzed using ImageJ. All antibodies have been tested for cross-reactivity and corresponding empty Vector control strain at the same generation and developmental stage was used parallelly as a negative control.

### Pull-down assay

100 μg of total protein lysate in 400μl of Pierce IP Lysis Buffer (see above) was mixed with 0.1 mg of Pierce Anti-HA Magnetic Beads (Thermo Scientific) and incubated for 2 hours at 4°C with gentle agitation. Samples were processed according to manufacturer’s protocol with few modifications allowing to remove remaining detergent for Mass Spectrometry analysis. In brief, additional 3 washing steps with 1 x TBS (1^st^ Base) were added after recommended wash with TBS-T (1x TBS, 0.1% Tween 20). For western blot analysis proteins were liberated from magnetic beads using acidic conditions (0.1M glycine (BioRad), pH 2.0) and neutralized with 1M Tris (pH 8.5) (1^st^ Base).

### Mass spectrometry sample preparation and analysis

HA-tagged magnetic beads from pull-down assay (see above) were washed 3x with 1 mL of TBS (1^st^ Base), resuspended in 100 μL of 8M Urea (BioRad) in 50mM Tris pH 8.0, then subsequently reduced with 20mM Tris(2-carboxyethyl)phosphine hydrochloride (Thermo Scientific) at 25°C for 20min, alkylated with 55mM chloroacetamide (Sigma) at room temperature in dark for 30 min. Samples were then diluted with 100mM TEAB to < 2M Urea and digested (4 hours, 25°C, shaking) with 1AU of Lysyl Endopeptidase (Lys-C) (FUJIFILM Wako Pure Chemical Corporation) and for 18 hours with 0.25 μg of Trypsin (Promega) (shaking, 25°C). To quench reaction samples were acidified with trifluoracetic acid to final concentration of 1% and further diluted with 2x the volume of 0.5% acetic acid. Peptides were loaded on activated Oasis HLB cartridges (Waters), washed twice with 2mL of 0.5% acetic acid and eluted with 1 mL of 50% acetonitrile in 0.5% acetic acid. Peptide concentration was quantified with Pierce Quantitative Fluorometric Peptide Assay (Thermo Scientific) and samples were dried in Speedvac (Eppendorf), resolubilized to 1 μg/μl and 1 μg from each sample was analyzed by nano-LC-MS/MS (Q-exactive HF, Thermo Scientific) on a 60 min linear gradient of mobile phase A (0.1% formic acid) and mobile phase B (acetonitrile, 0.1% formic acid). Raw data was analyzed in Proteome Discoverer 2.5 (Thermo Scientific) using label-free quantitation algorithm for quantitation of precursor ions. SEQUEST database was used to search MS/MS spectra against combined human (Uniprot) and P. falciparum (PlasmoDB) protein databases. Data analysis settings included up to 3 missed tryptic cleavages, carbamidomethylation (C) as a static modification and Oxidation (M), Deamination (N,Q), Acetylation (N-terminus) as dynamic modifications. Mass error tolerance of 20ppm and 0.05Da was used for precursor ion and fragment mass tolerance respectively. Quantitative data was further normalized across the samples using total peptide amount. Abundances were scaled for each protein where total arbitrary value of 600 has been assigned to all samples per each developmental stage (rings, trophozoites and schizonts) and divided accordingly between all samples (3x PfCYP19B-OE and 3x empty vector controls) based of the signal of the protein in each replicate. Samples where protein has not been present were assigned value 1 to avoid division by 0. Ratio of empty Vector control signal (background) to *Pfcyp19b*-OE signal was calculated. Accordingly, ratio of 200 means that no traces of the protein were detected in background control replicates. Binding candidates in Table 1 were picked based on the ratio >3 and number of peptide spectrum matches (PSM) >3.

### Generation of luciferase constructs

Dual Luciferase Assay was performed using previously published methods with slight modifications to the protocols [67,68]. In brief, panel of four constructs encompassing entire intergenic region between PfCYP19B and PfFP2A was de novo synthetized by Bio-basic Asia (Singapore). Each construct contained one of the deletions analyzed in this study located 426bp upstream of the start codon. Deletions were labeled as ^del^(10xAT), ^del^(7xAT), ^del^(6xAT), the wild type 3D7 reference (^ref^3D7) sequence (no deletion) and visualized in **Fig 4A**. Constructs were flanked with Ncol Xhol restriction sites, cloned into *E*. *coli*, amplified, and validated through capillary sequencing Bio-basic Asia (Singapore). Correct constructs were subsequently ligated into pPf86 plasmid backbone designed previously [68]. Pfhps86 promoter originally located upstream of the luciferase gene (FL) was removed using NcoI and XhoI restriction enzymes (New England Biolabs) and replaced with the synthetized constructs (Bio-basic Asia, Singapore). Plasmids with correct sequences were transformed into DH5α *E*. *coli* competent cells and colonies were grown on LB agar (+ampicillin) for 24 hours. One colony from each construct was selected and further grown in Terrific Broth (BD) for 24 hours. Plasmids were extracted using Giga Prep Plasmid kit (QIAGEN) to generate enough plasmid material for all subsequent assays. All constructs were additionally verified at this step using Sanger sequencing to control for any DNA replication errors that could occur in bacteria. Plasmid concentrations were measured on Qubit dsDNA BR assay and its concentrations normalized between samples. Final concentrations have been verified through additional Qubit dsDNA BR assay and qPCR.

### Transient transfection of *P*.* falciparum* parasites and dual-luciferase reporter assays

5 0μg of previously designed luciferase constructs (above) containing various AT deletions in *Pfcyp19b* upstream intergenic region (Fig 4) were transfected into 6 independently grown populations of desynchronized 3D7 MR4 parasites at 15% parasitemia by electroporation as described [67,68]. All parasite samples were also simultaneously transfected with 50 μg of the plasmid expressing renilla luciferase (RL) which was used as reference for normalizing firefly luciferase (FL) activity (total 100μg of plasmid per transfection). Original pPf86 plasmid with Pfhps86 promoter was used as a positive control. Transfection with no plasmid was used as a negative control for the background luminescence. After electroporation parasites we placed in prewarmed RPMI media. The flasks were gassed and maintained in culture for 4 hours after which RPMI media has been changed and parasites were grown for additional 24 hours until harvest. Parasites were liberated from RBCs with 0.1% saponin as described above. The Dual Luciferase Reporter Assay system (Promega) was used as described by manufacturer to determine both the firefly (target expression) and the renilla (reference expression) luciferase activities. The luciferase readings for the negative control (without plasmid) were used to determine background auto-luminescence levels. Firefly luciferase and renilla luciferase activity (luminescence) were measured in relative light units (RLUs) for 10 seconds on Infinite 200 Pro Multimode Plate Reader (TECAN). Normalized luciferase activity was calculated by the following formula: “FLuc_normalized_ = (FLuc–Fluc_bg_)/(RLuc–Rluc_bg_)” where FLuc is firefly luciferase and RLuc is renilla luciferase, and bg stands for the background. All transfection experiments were performed in 6 independently grown biological replicates.

### Real-time PCR (qPCR)

Levels of plasmid DNA material were estimated in each sample by qPCR. Real time PCRs and the relative quantification of gene expression were performed in triplicates using the comparative CT method of calculating -ΔΔCt values and taking arginase tRNA as an internal reference control gene, as previously described [93].

### Cyclophilin locus amplification and amplicon sequencing

To investigate the genetic variations in the proximity of *Pfcyp19b* gene locus, we designed a pair of primers (F/GGTTCCAGAACCTGATAAAGAACCTGATAATG, R/GCAACCAAACCTAGTAATAATGGTCCAGTAAG) to capture and amplify 18.7Kb genomic region flanking *Pfcyp19b* region with adjacent genes (FP2B, FP3, Api-AP2, FP2A, PF3D7_1115800 –location: *PF3D7_11*. *578*,*385*.. *597*,*119*) (**S9 Fig**). Primers were *in silico* and *in vitro* tested for specificity against human and *P*. *falciparum* gDNA material. Fragment was amplified using 0.63U (0.5ul) of very high-fidelity ultra-long-range DNA polymerase PrimeSTAR GLX (Takara). 2 ng of extracted, purified gDNA from 144 TRAC2 clinical samples (see above) was subjected to 30 cycles of PCR with following conditions: 98°C/10 sec; 66°C/20 min (1 min/kb); 4°C/hold, 0.2 μM of each primer in 25 μl total reaction volume. To prevent evaporation due to very long annealing times plates were sealed firmly with highly adhesive transparent seals (Thermo Fisher) and amplified in the thermal cycler with flexible silicon heated lid (Eppendorf). PCR product was purified using 0.6 volumes (v/v) of AMPure XP beads (Beckman Coulter). Resulted amplicons were processed using Nextera XT library prep kit (Illumina) to obtain short sequence reads according to manufacturer instructions with minor modifications. In brief. 0.25 ng of amplicon DNA was tagmented and amplified using downscaled library preparation protocol with 1/4 volume of recommended reagents being used. PCR amplification cycles were increased from 12 to 15 cycles. PCR reactions were purified using 0.6 volumes (v/v) of AMPure XP magnetic beads following manufacturer’s instructions. Samples were eluted in 17 μl of 10 mM Tris-Cl, pH 8.5 (Qiagen) and average library sizes were estimated on Bioanalyzer High Sensitivity chips (Agilent). Library molarity was assessed with qPCR and libraries were pooled and sequenced on one lane of Novaseq HiSeq platform (Illumina) with pair-end 150bp long reads protocol, generating approximately 20 GB of data (Novogene Co., Singapore).

### Amplicon sequencing analysis

Reads were trimmed using Trim Galore! package [94] before alignment by BWA [95]. Further, PCR duplicates were removed using Picard tools [96] and bases were recalibrated by GATK BQSR [97]. Short variants form the targeted regions were called with GATK Haplotypecaller [97]. Variants were filtered by GATK hard filters specifying arguments: “MQ <60 or FS >60 or QD <2.0”. Bi-allelic SNPs and Indels were re-coded in binary(presence/absence), whereas multi-allelic insertion/deletion variations were re-coded as loss or gain of bases and associated with *Pfcyp19b* gene expression through linear regression. Next, genome sequences were downloaded in BAM format from TRACI and TRACII study database with overlapping transcriptome data [5,87]. Short variants were identified and filtered using method described above. Amplicon sequencing raw data are available in fastq format submitted to NCBI sequence read archive under BioProject PRJNA871873 (available from: https://www.ncbi.nlm.nih.gov/bioproject/PRJNA871873)

### Statistical analyses

Statistical analyses were carried out using GraphPad Prism v8.4.2 (GraphPad Software Inc., USA) and/or Microsoft Excel 365 (Microsoft Co., USA) or as described in Amplicon sequencing analysis section.

## Supporting information

S1 Fig Microarray-based *pfcyp19b* expression levels to PC1/2 from TRACI and TRACII. A) Scatter plot showing microarray-based *pfcyp19b* expression levels (ratio to the reference strain, *log2*) in relation to PC1/2 in parasite samples collected during TRACI study (2011–2013) [35] B) Scatter plot showing microarray-based *pfcyp19b* expression levels (ratio to reference strain, log2) to PC1/2 in parasite samples collected during TRACII study (2015–2018) [31]. Both scatter plots show a positive linear correlation between transcript levels of *pfcyp19b* and the parasite clearance half-life (TRACI: PCC = 0.21, TRACII: PCC = 0.11). *Pfcyp19b* appears transcriptionally upregulated in *P*. *falciparum* collected from slow-clearing infections (PC1/2 > 5hr, TRACI *p* = 6.36E-08 and TRACII *p* = 0.004, unpaired two-tailed t-test).
(TIF)

S2 Fig *In vitro pfcyp19b* expression values in pfk13 mutants across parasite’s IDC. To investigate the potential link between *pfk13* mutation and *pfcyp19b* expression we have extracted data from previously published *in vitro* study showing *pfcyp19b* expression levels throughout the entire IDC in several *pfk13* mutant strains–on the background of commonly used Dd2 line and field-derived Cambodian isogenic Cam3.II lines [37]. Notably, Dd2 strain has been adapted to laboratory culture in the 1980s, long before ACTs were introduced as anti-malarial therapy in SE Asia while Cam3.II line was culture-adapted almost a decade after ACT introduction. (A-C) Averaged *pfcyp19b* expression values in *pfk13* mutants (C580Y, R539T) generated under distinct genetic backgrounds of Cam3.II lines (A) and Dd2 (B-C). *Pfcyp19b* peak expression values fall during the schizont stage. *Pfk13* expression values are shown in gray with its expression peaking at schizont/ring transition time (48/0 HPI). Our analysis showed nearly 2-fold *pfcyp19b* upregulation at the late schizont to early rings transition stage (48 HPI / 0 HPI) in the isogenic Cam3.II R539T Cambodian strain when compared Cam3.II wild type (average *log2* expression increase at 0HPI = 0.85, at 48HPI = 0.98). This specific early-stage upregulation stands in agreement with our earlier observation of *in vivo* field parasites (*see Fig 1A*). Interestingly, DD2 mutants (both C580Y and R539T) did not show any significant *pfcyp19b* upregulation, suggesting that this might be background specific. Curiously, when projected against the expression of *pfk13 (gray lines)*, *pfcyp19b* appears to be mutually exclusive and the upregulation of *pfcyp19b* in Cam3.II R539T is limited only to the stages with peak expression of *pfk13*. All values are derived from microarray assays and are shown as a ratio to microarray reference pool expression (l*og2*). Error bars show standard deviation. The X-axis shows the estimated parasite age. (D) Median PfCYP19B protein fold change increase in *pfk13* Cam3.II mutants compared to the wild type control from two independent experiments at the ring stage. There was a marked difference in PfCYP19B protein levels which was significantly higher in both Cam3.II C580Y and R539T lines. Asterixes indicate statistical significance based on a two-tailed heteroscedastic t-test on the *log2* normalized peptide spectral intensities, when comparing a *pfk13* mutant against its isogenic wild-type counterpart; * *p* < 0.05, ** *p* < 0.01, *** *p* < 0.001, **** p < 0,0001. Error bars indicate standard deviation.
(TIF)

S3 Fig Western blot validation of continuous PfCYP19B overexpression. Above: western blot image showing continuous episomal overexpression of PfCYP19B-3xHA protein across the entire IDC of the parasite. Below: western blot image showing increased expression of total PfCYP19B protein in all three distinct parasite IDC stages.
(TIF)

S4 Fig Ring survival assays performed on *Pfcyp19b*-OE. Ring survival assay performed on *Pfcyp19b*-OE and negative empty Vector control. Significance is based on the results obtained from five independent biological replicates and unpaired two-tailed heteroscedastic t-test with Holm-Sidak correction.
(TIF)

S5 Fig Drug susceptibility assays in the presence of CsA. Drug susceptibility assays on *Pfcyp19b*-OE (left) and Empty Vector control (right) strains against various antimalarial compounds in the absence (black) or presence (gray) of 80 nM cyclosporine A (CsA). Error bars indicate the standard deviation of the IC_50_ in each treatment conducted in 3 independent biological replicates. Asterixes indicate statistical significance based on paired two-tailed heteroscedastic t-test where: * *p* < 0.05, ** *p* < 0.01.
(TIF)

S6 Fig Effect of *pfcyp19b* overexpression on CsA sensitivity. Cyclosporine A (CsA) drug susceptibility assays on *Pfcyp19b*-OE and Empty Vector control strains. Error bars indicate the standard deviation of the IC_50_ in each treatment conducted in 3 independent biological replicates. Numbers above show averaged IC_50_ values.
(TIF)

S7 Fig PfeIF2α-P levels after gradual PPQ treatments. Western blot analysis of PfeIF2α-P levels under 500 nM DHA stress in ring parasites simultaneously co-treated with gradually decreasing concentrations of PPQ (n = 1). Parasites were treated either only with 500 nM DHA or the combination of DHA and PPQ in a 10-fold dilution gradient. Graph (left) shows PfeIF2α-P level fold change between DHA/PPQ treated parasites and DHA treatment alone. All values have been normalized to PfBiP internal loading control. The gel image is shown on the right. DMSO has been used as a negative control to indicate PfeIF2α-P baseline levels.
(TIF)

S8 Fig Effect of CsA treatment on PfeIF2-α phosphorylation levels in PfCYP19B overexpressing parasites. Western blot analysis showing alleviating effect of PfCYP19B overexpression (*Pfcyp19b*-OE) on PfeIF2α-P levels after ER stress induced by 90 min 4 μM CsA treatment. The experiment was performed on ring stage parasites episomally overexpressing PfCYP19B (*Pfcyp19b*-OE) and empty Vector control line (n = 1). All values were normalized to PfBiP internal loading control.
(TIF)

S9 Fig Illustration of *pfcyp19b* locus region used for amplicon sequencing. Graphical representation of genomic DNA fragment from chromosome 11 used for amplicon sequencing of TRACII samples shown in Fig 4. The entire region between two blue dashed vertical lines has been amplified and sequenced on the Illumina NovaSeq platform. The location of 426 eSTR at position 591054 has been indicated by the black arrow. Distances are approximately scaled only for illustrative purposes. PF3D7_1115300—cysteine proteinase falcipain 2b, PF3D7_1115400—cysteine proteinase falcipain 3, PF3D7_1115500—AP2 domain transcription factor, PF3D7_1115600—peptidyl-prolyl cis-trans isomerase, PF3D7_1115700—cysteine proteinase falcipain 2a
(TIF)

S10 Fig Plasmids used in this study. Schematic representation of plasmids used to generate *Pfcyp19b*-OE (top), empty Vector control (middle), and GFP/FKBP-tagged PfCYP19B strain (bottom).
(TIF)

S1 Data *PfCyp19b*-OE Pull-down results. Unprocessed results showing mass spectrometry hits from HA-tagged PfCYP19B pull-down assay.
(XLSX)

S2 Data Short Tandem Repeats genotyping results. All short tandem repeats polymorphic variants called from examined cyclophilin locus from TRACI WGS, TRACII WGS, and TRACII AmpliSeq datasets.
(XLSX)

S3 Data Source data. Source data used to generate all figures in this study
(XLSX)

S1 Images Western blot images. Full unprocessed western blot images used in the study.
(PDF)
