# Supplementary figures and images for: Short tandem repeat polymorphism in the promoter region of cyclophilin 19B drives its transcriptional upregulation and contributes to drug resistance in the malaria parasite Plasmodium falciparum
Source: PLoS Pathog. 2023 Jan 25;19(1):e1011118. doi: 10.1371/journal.ppat.1011118 (PMC9901795; doi:10.1371/journal.ppat.1011118)

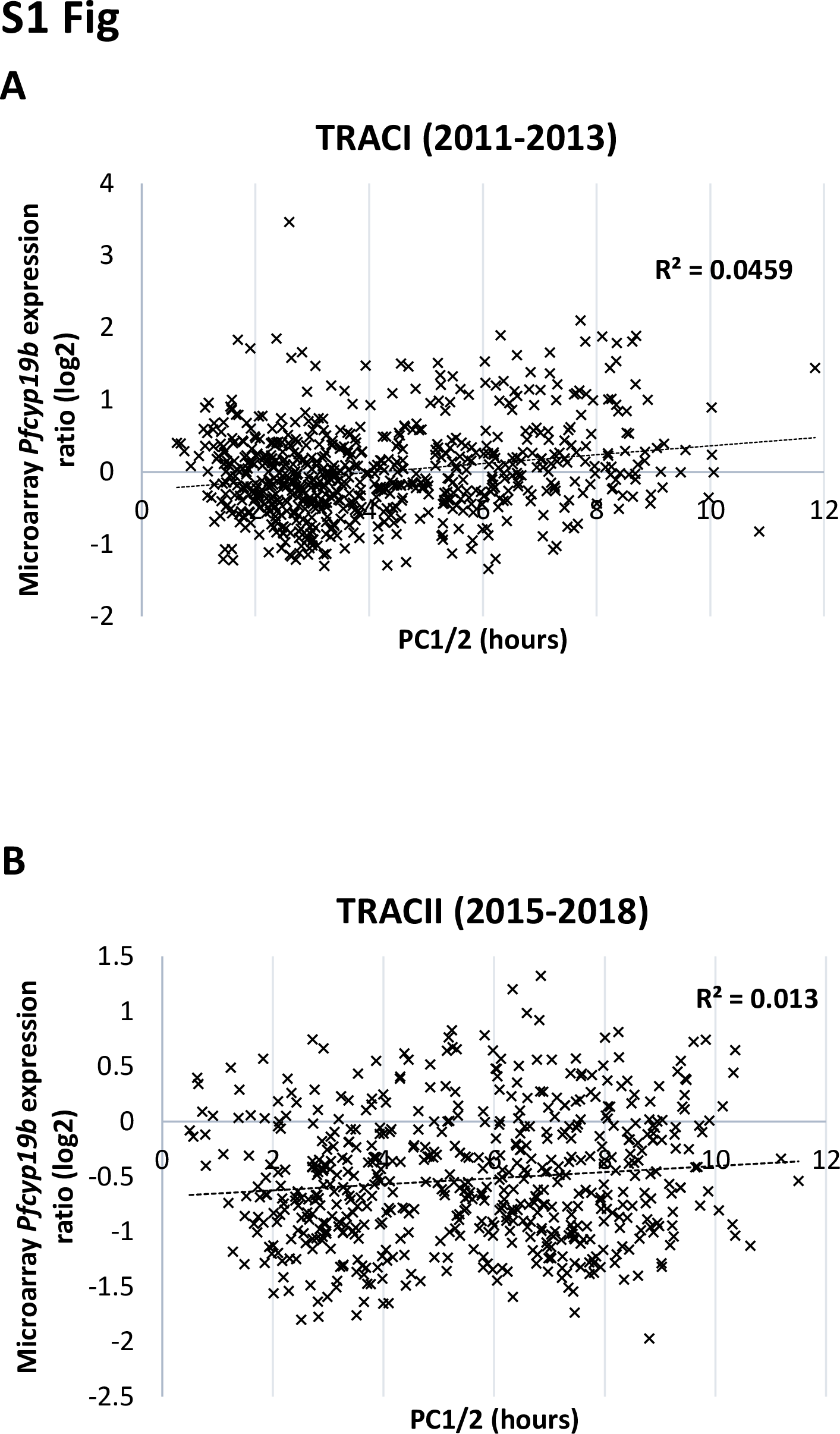

Supplement: S1 Fig — A) Scatter plot showing microarray-based pfcyp19b expression levels (ratio to the reference strain, log2) in relation to PC1/2 in parasite samples collected during TRACI study (2011–2013) [35] B) Scatter plot showing microarray-based pfcyp19b expression levels (ratio to reference strain, log2) to PC1/2 in parasite samples collected during TRACII study (2015–2018) [31]. Both scatter plots show a positive linear correlation between transcript levels of pfcyp19b and the parasite clearance half-life (TRACI: PCC = 0.21, TRACII: PCC = 0.11). Pfcyp19b appears transcriptionally upregulated in P. falciparum collected from slow-clearing infections (PC1/2 > 5hr, TRACI p = 6.36E-08 and TRACII p = 0.004, unpaired two-tailed t-test). (TIF) [file ppat.1011118.s001.tif]

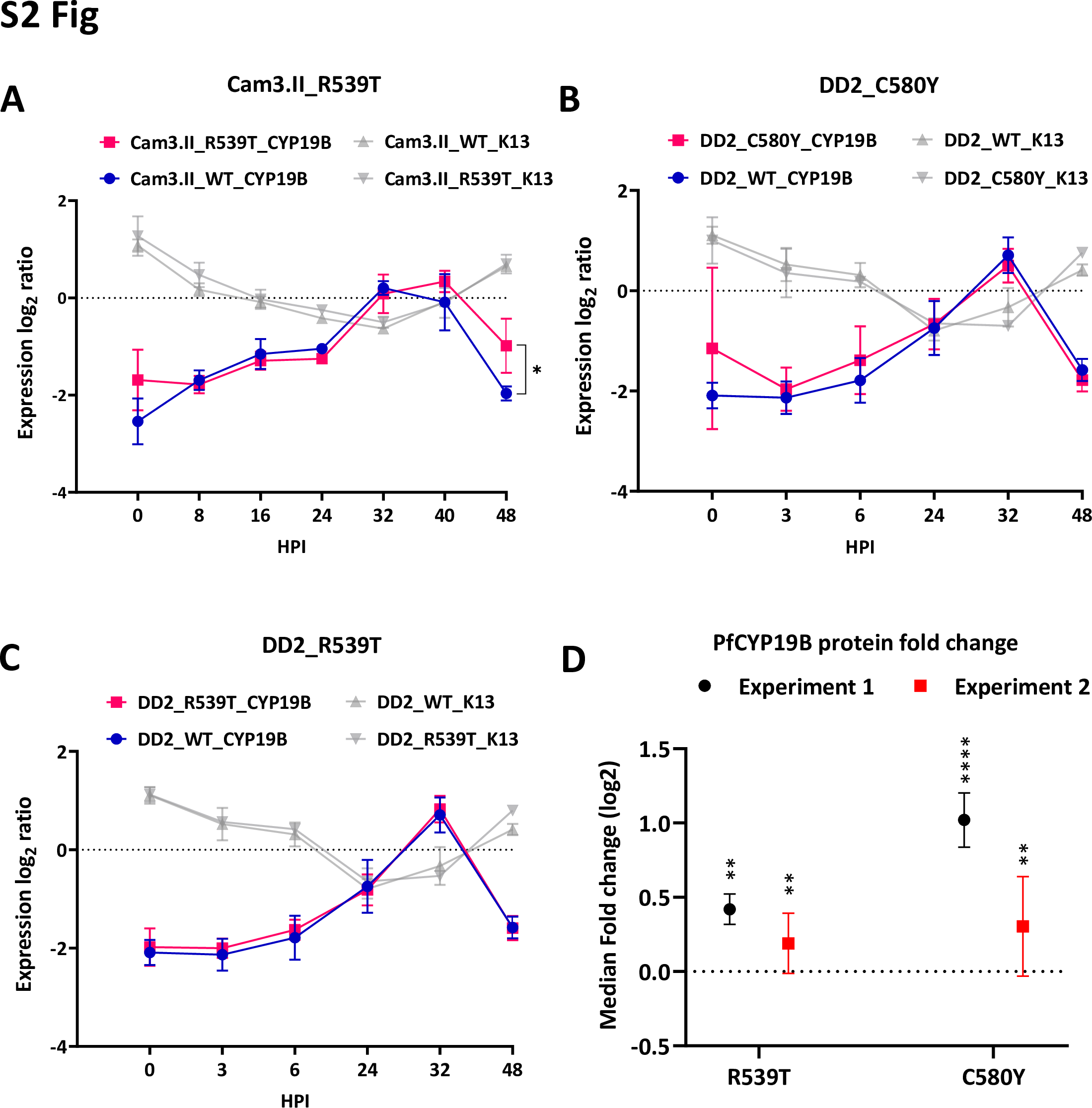

Supplement: S2 Fig — To investigate the potential link between pfk13 mutation and pfcyp19b expression we have extracted data from previously published in vitro study showing pfcyp19b expression levels throughout the entire IDC in several pfk13 mutant strains–on the background of commonly used Dd2 line and field-derived Cambodian isogenic Cam3.II lines [37]. Notably, Dd2 strain has been adapted to laboratory culture in the 1980s, long before ACTs were introduced as anti-malarial therapy in SE Asia while Cam3.II line was culture-adapted almost a decade after ACT introduction. (A-C) Averaged pfcyp19b expression values in pfk13 mutants (C580Y, R539T) generated under distinct genetic backgrounds of Cam3.II lines (A) and Dd2 (B-C). Pfcyp19b peak expression values fall during the schizont stage. Pfk13 expression values are shown in gray with its expression peaking at schizont/ring transition time (48/0 HPI). Our analysis showed nearly 2-fold pfcyp19b upregulation at the late schizont to early rings transition stage (48 HPI / 0 HPI) in the isogenic Cam3.II R539T Cambodian strain when compared Cam3.II wild type (average log2 expression increase at 0HPI = 0.85, at 48HPI = 0.98). This specific early-stage upregulation stands in agreement with our earlier observation of in vivo field parasites (see Fig 1A). Interestingly, DD2 mutants (both C580Y and R539T) did not show any significant pfcyp19b upregulation, suggesting that this might be background specific. Curiously, when projected against the expression of pfk13 (gray lines), pfcyp19b appears to be mutually exclusive and the upregulation of pfcyp19b in Cam3.II R539T is limited only to the stages with peak expression of pfk13. All values are derived from microarray assays and are shown as a ratio to microarray reference pool expression (log2). Error bars show standard deviation. The X-axis shows the estimated parasite age. (D) Median PfCYP19B protein fold change increase in pfk13 Cam3.II mutants compared to the wild type control from two independe [file ppat.1011118.s002.tif]

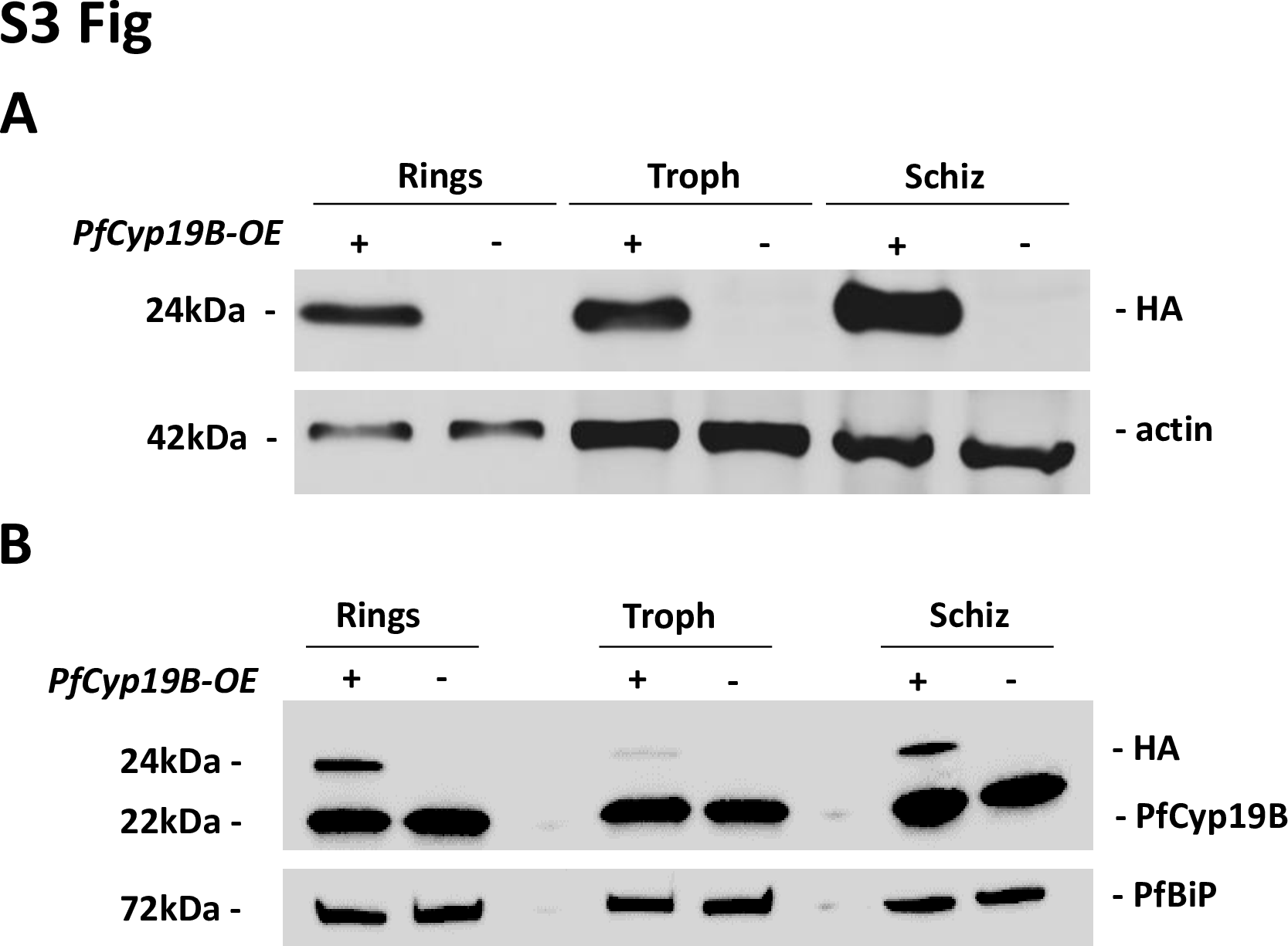

Supplement: S3 Fig — Above: western blot image showing continuous episomal overexpression of PfCYP19B-3xHA protein across the entire IDC of the parasite. Below: western blot image showing increased expression of total PfCYP19B protein in all three distinct parasite IDC stages. (TIF) [file ppat.1011118.s003.tif]

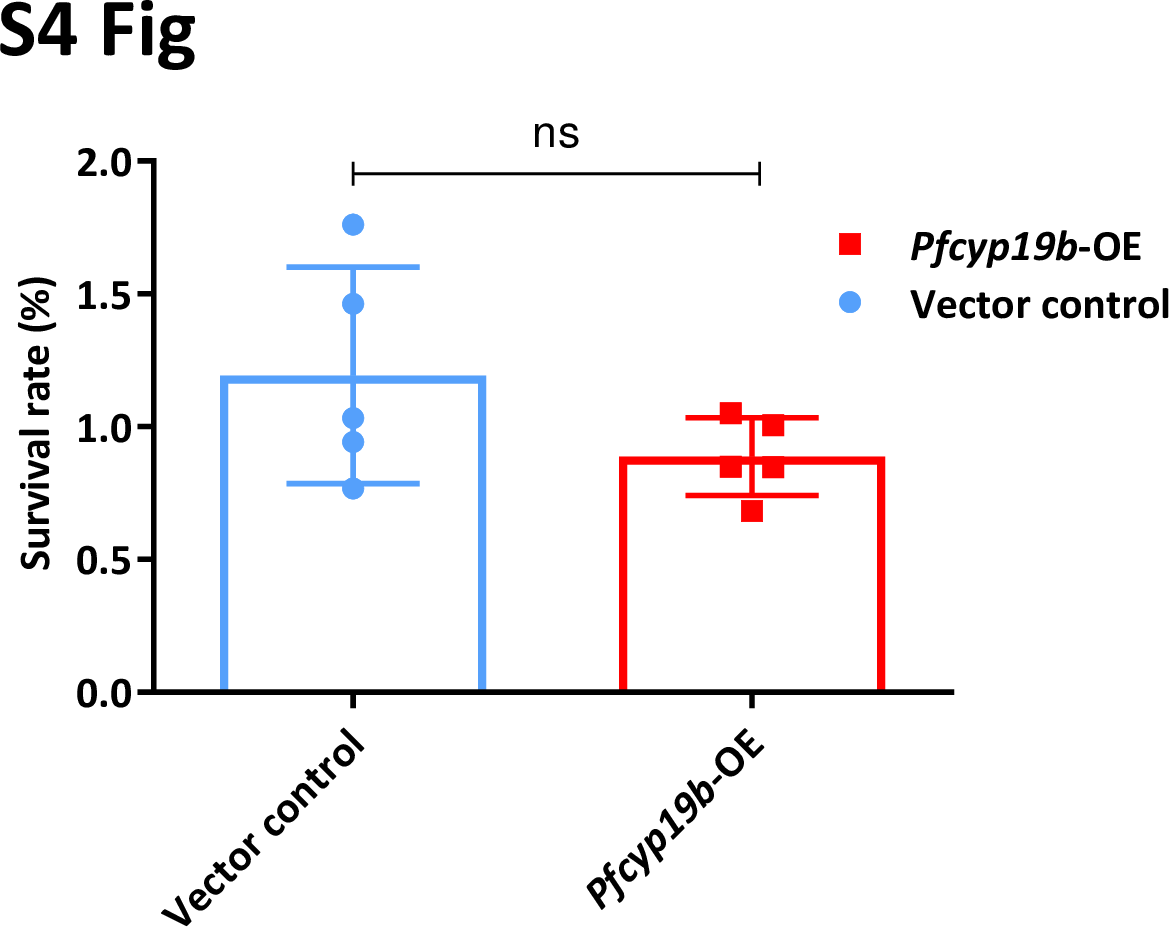

Supplement: S4 Fig — Ring survival assay performed on Pfcyp19b-OE and negative empty Vector control. Significance is based on the results obtained from five independent biological replicates and unpaired two-tailed heteroscedastic t-test with Holm-Sidak correction. (TIF) [file ppat.1011118.s004.tif]

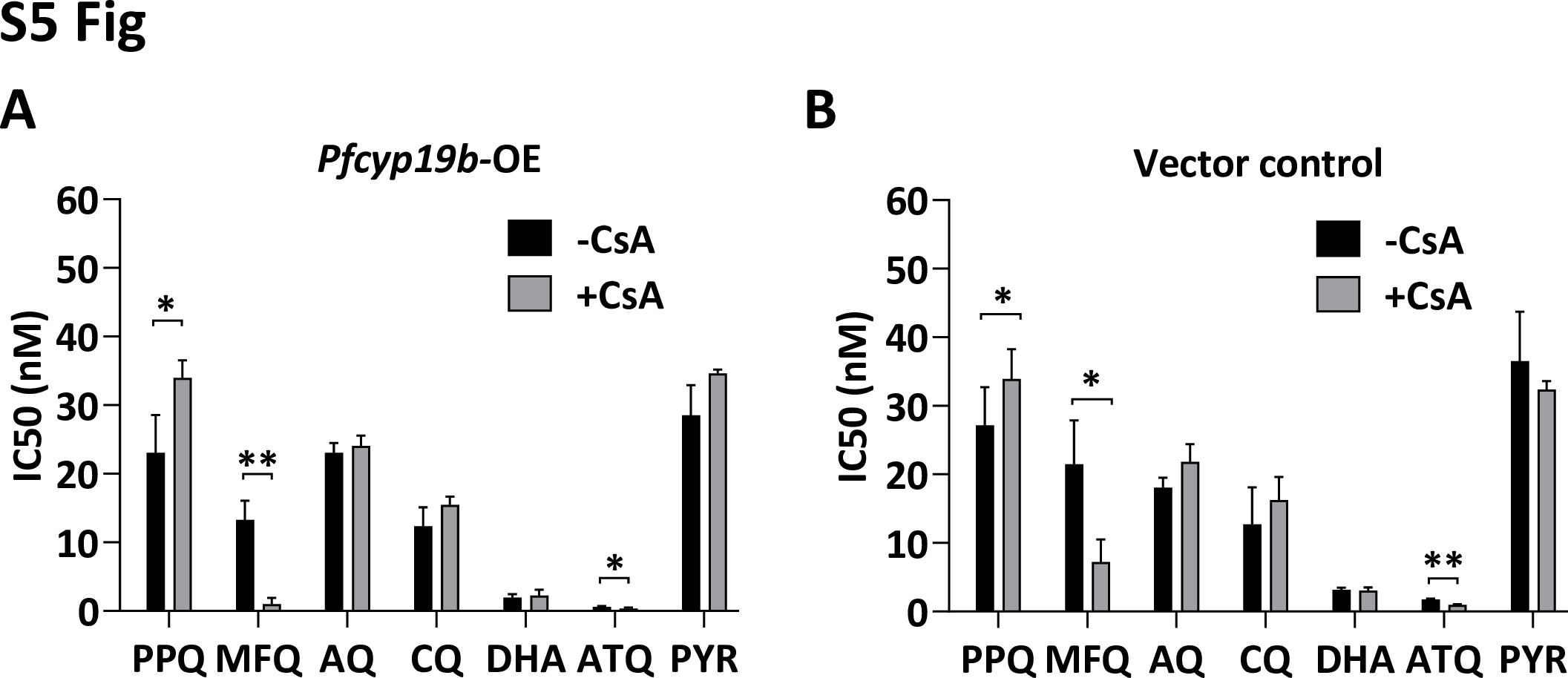

Supplement: S5 Fig — Drug susceptibility assays on Pfcyp19b-OE (left) and Empty Vector control (right) strains against various antimalarial compounds in the absence (black) or presence (gray) of 80 nM cyclosporine A (CsA). Error bars indicate the standard deviation of the IC50 in each treatment conducted in 3 independent biological replicates. Asterixes indicate statistical significance based on paired two-tailed heteroscedastic t-test where: * p < 0.05, ** p < 0.01. (TIF) [file ppat.1011118.s005.tif]

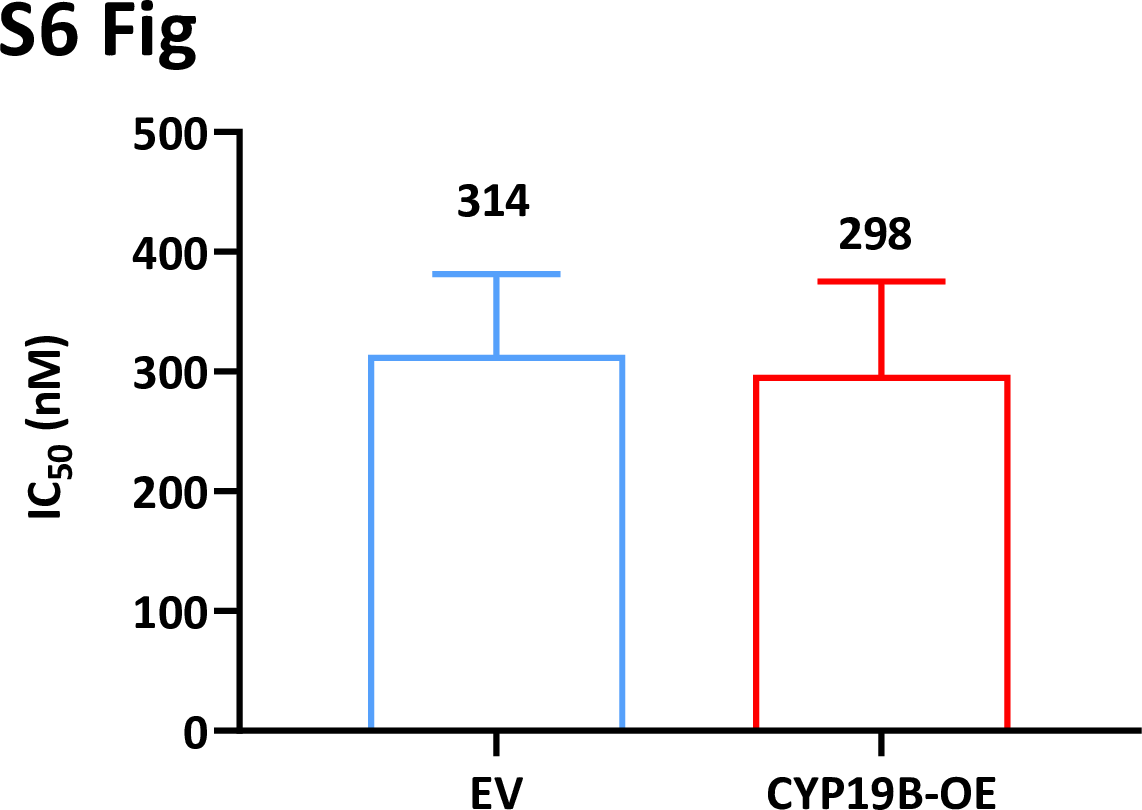

Supplement: S6 Fig — Cyclosporine A (CsA) drug susceptibility assays on Pfcyp19b-OE and Empty Vector control strains. Error bars indicate the standard deviation of the IC50 in each treatment conducted in 3 independent biological replicates. Numbers above show averaged IC50 values. (TIF) [file ppat.1011118.s006.tif]

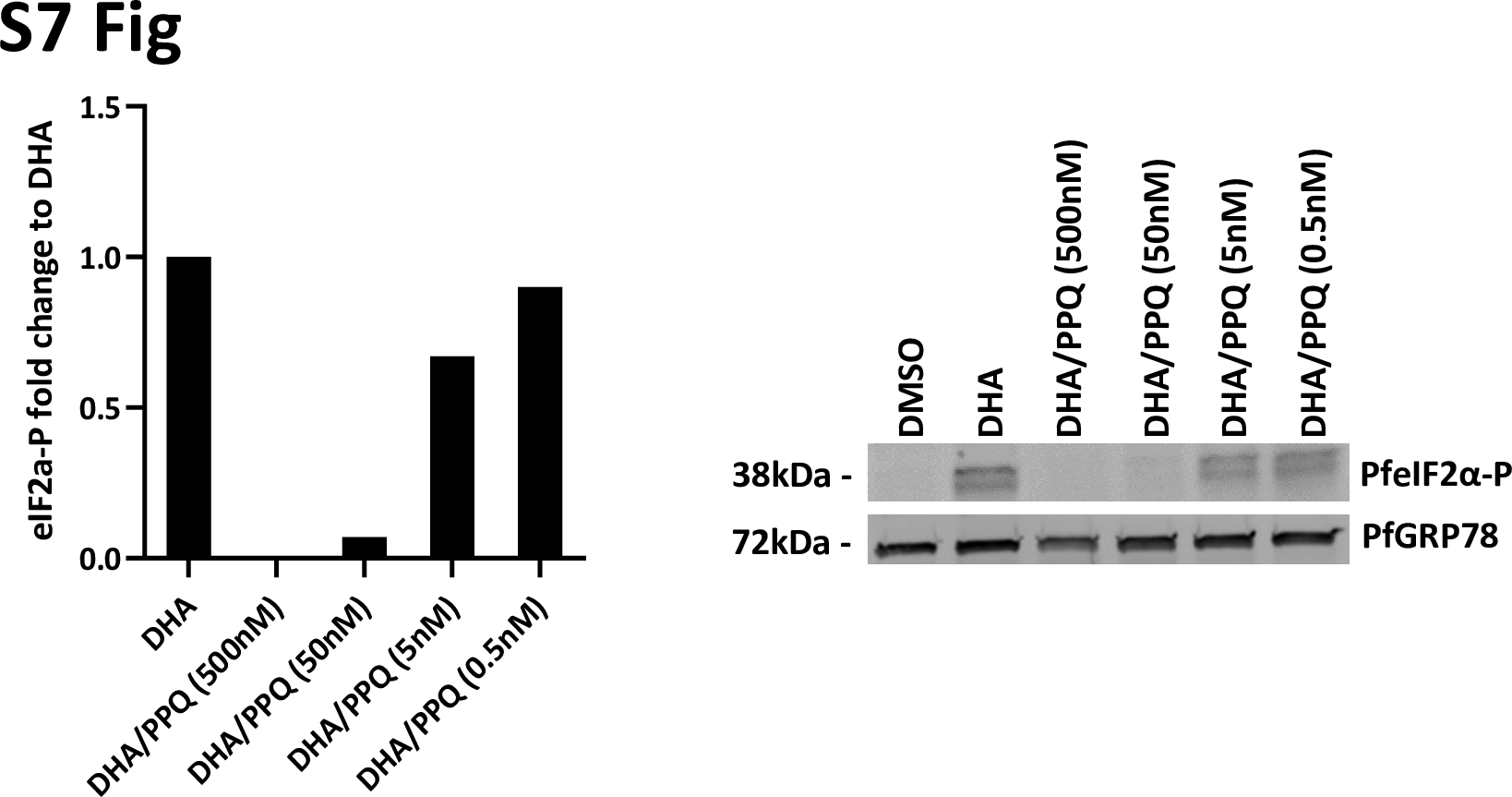

Supplement: S7 Fig — Western blot analysis of PfeIF2α-P levels under 500 nM DHA stress in ring parasites simultaneously co-treated with gradually decreasing concentrations of PPQ (n = 1). Parasites were treated either only with 500 nM DHA or the combination of DHA and PPQ in a 10-fold dilution gradient. Graph (left) shows PfeIF2α-P level fold change between DHA/PPQ treated parasites and DHA treatment alone. All values have been normalized to PfBiP internal loading control. The gel image is shown on the right. DMSO has been used as a negative control to indicate PfeIF2α-P baseline levels. (TIF) [file ppat.1011118.s007.tif]

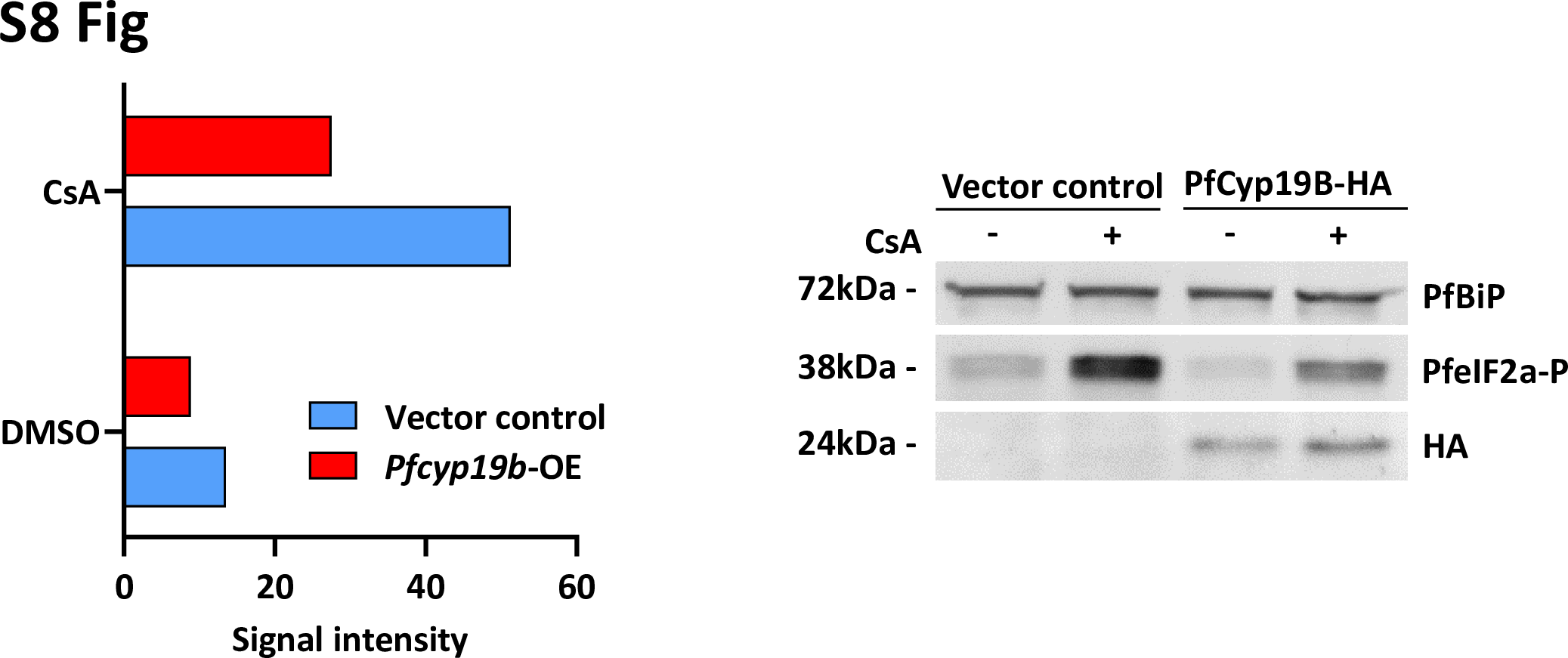

Supplement: S8 Fig — Western blot analysis showing alleviating effect of PfCYP19B overexpression (Pfcyp19b-OE) on PfeIF2α-P levels after ER stress induced by 90 min 4 μM CsA treatment. The experiment was performed on ring stage parasites episomally overexpressing PfCYP19B (Pfcyp19b-OE) and empty Vector control line (n = 1). All values were normalized to PfBiP internal loading control. (TIF) [file ppat.1011118.s008.tif]

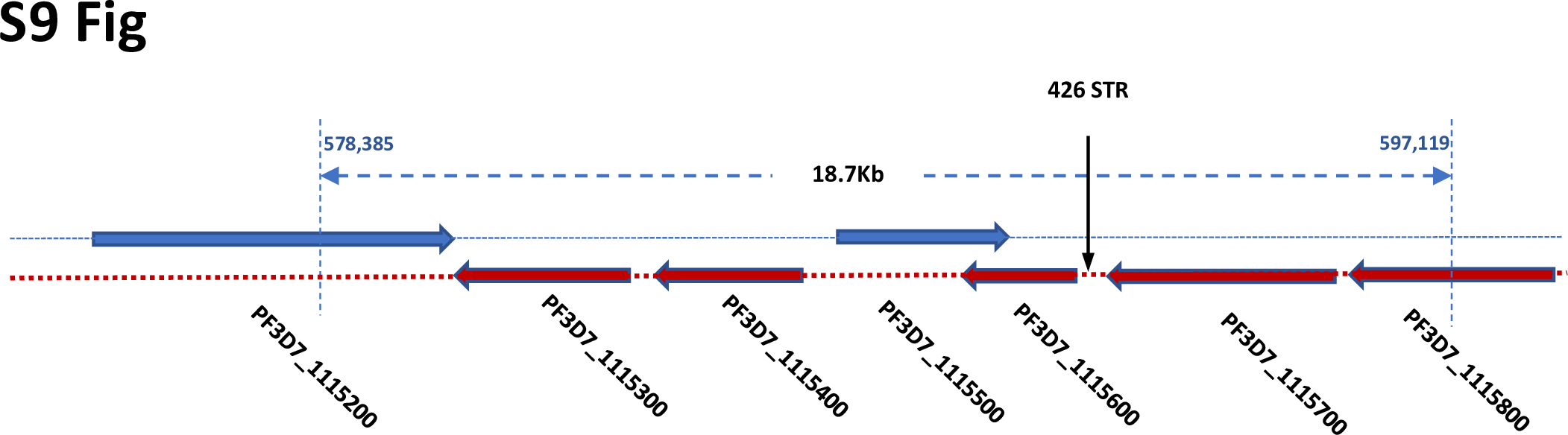

Supplement: S9 Fig — Graphical representation of genomic DNA fragment from chromosome 11 used for amplicon sequencing of TRACII samples shown in Fig 4. The entire region between two blue dashed vertical lines has been amplified and sequenced on the Illumina NovaSeq platform. The location of 426 eSTR at position 591054 has been indicated by the black arrow. Distances are approximately scaled only for illustrative purposes. PF3D7_1115300—cysteine proteinase falcipain 2b, PF3D7_1115400—cysteine proteinase falcipain 3, PF3D7_1115500—AP2 domain transcription factor, PF3D7_1115600—peptidyl-prolyl cis-trans isomerase, PF3D7_1115700—cysteine proteinase falcipain 2a (TIF) [file ppat.1011118.s009.tif]

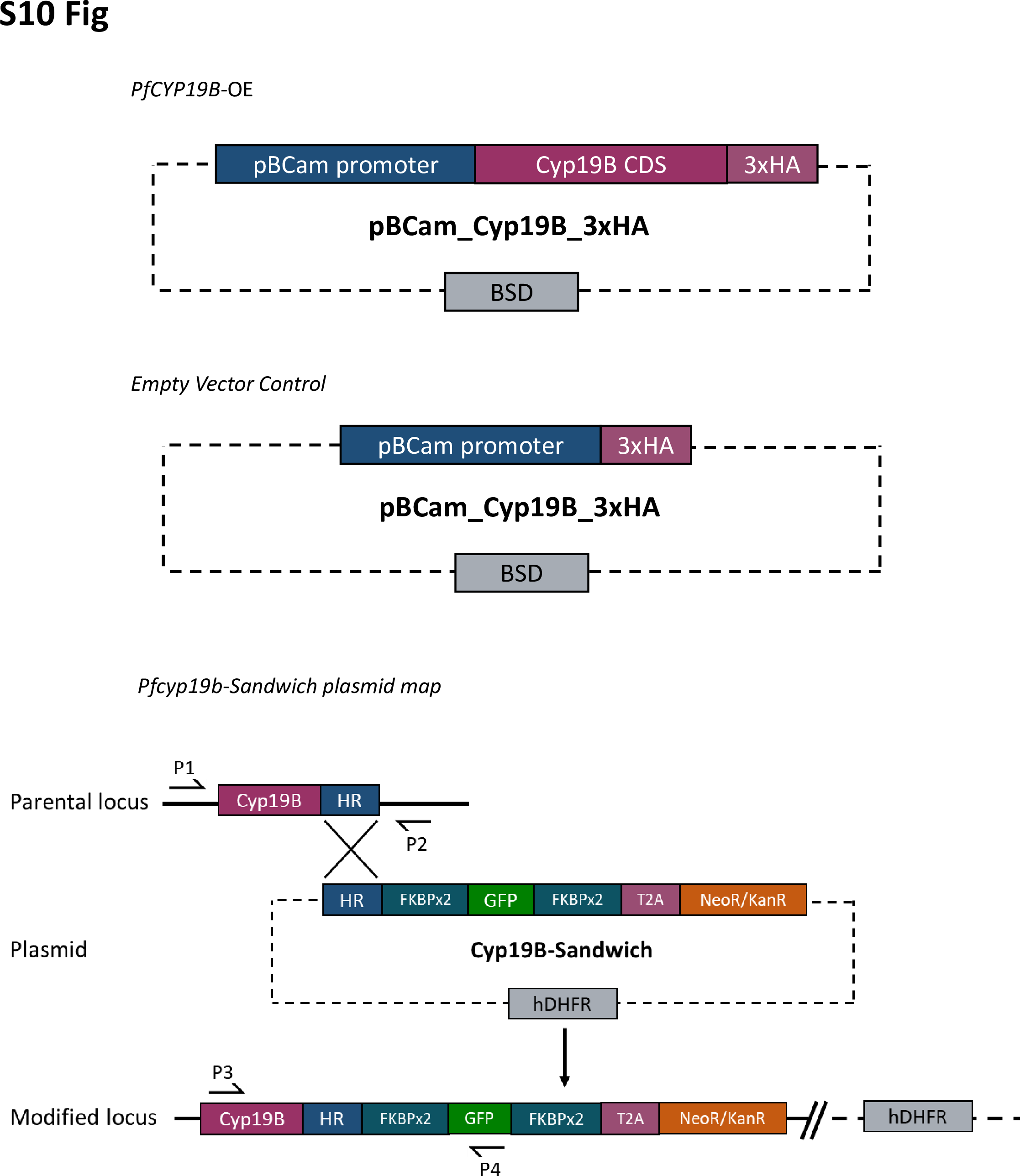

Supplement: S10 Fig — Schematic representation of plasmids used to generate Pfcyp19b-OE (top), empty Vector control (middle), and GFP/FKBP-tagged PfCYP19B strain (bottom). (TIF) [file ppat.1011118.s010.tif]
