# Supplementary material for: Short tandem repeat polymorphism in the promoter region of cyclophilin 19B drives its transcriptional upregulation and contributes to drug resistance in the malaria parasite Plasmodium falciparum
Source: PLoS Pathog. 2023 Jan 25;19(1):e1011118. doi: 10.1371/journal.ppat.1011118 (PMC9901795; doi:10.1371/journal.ppat.1011118)

Supplementary Images

Full unprocessed western blot images used in the study

a) Western Blots Fig 2A

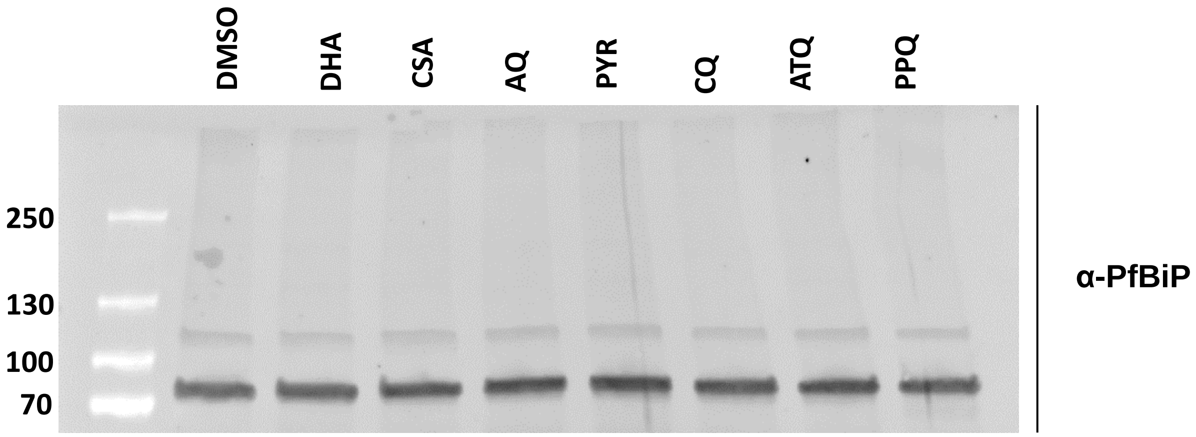

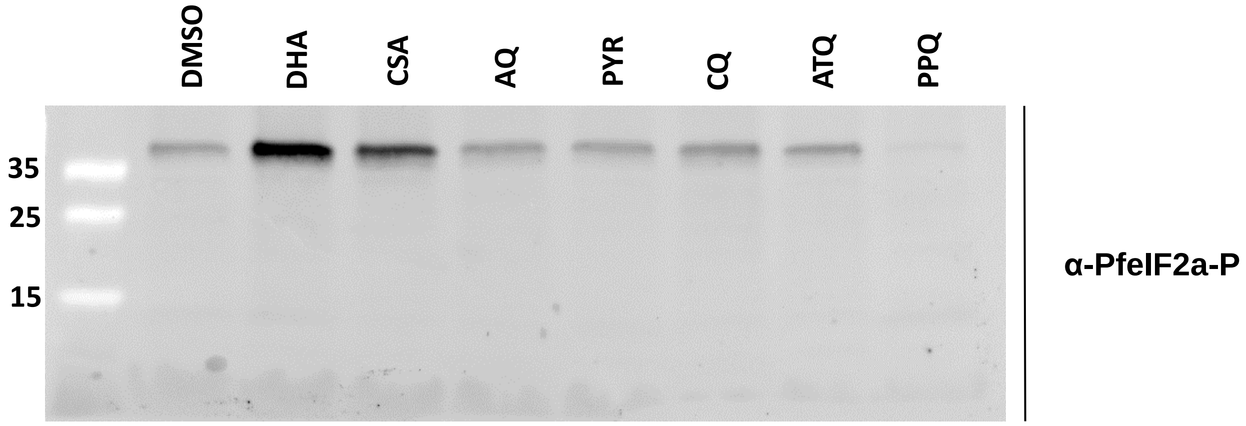

b) Western Blots Fig 2B

Replicate 1

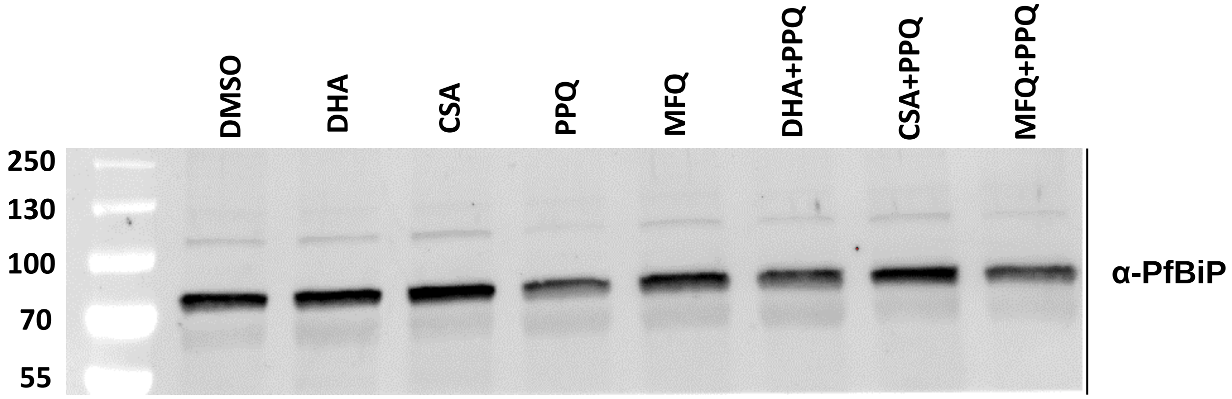

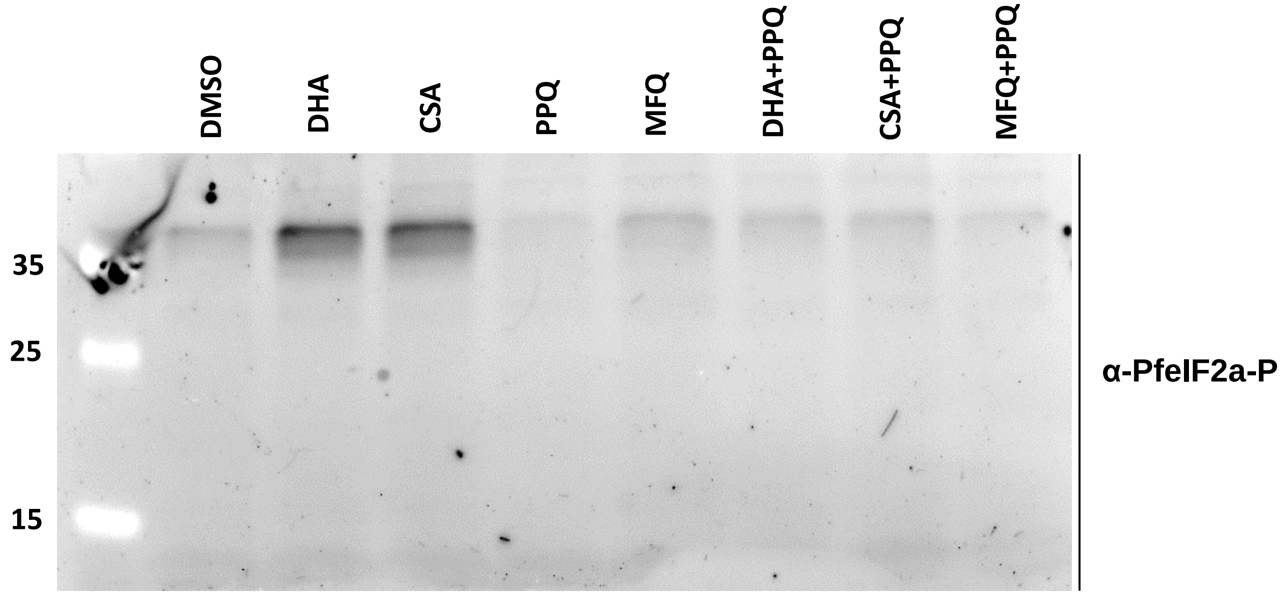

Replicate 2

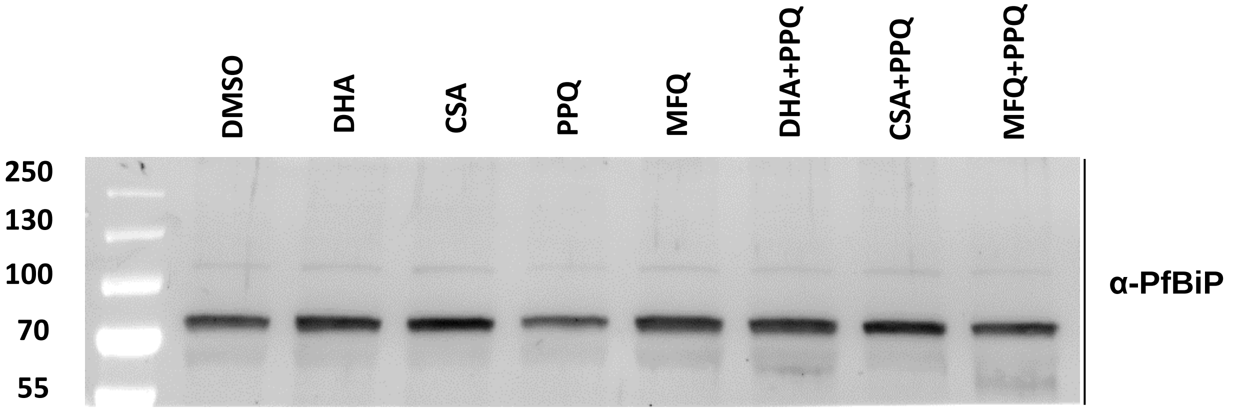

DMSO
DHA
CSA
PPQ
MFQ
DHA+PPQ
CSA+PPQ
MFQ+PPQ
250
130
100
70
55
α-PfBiP

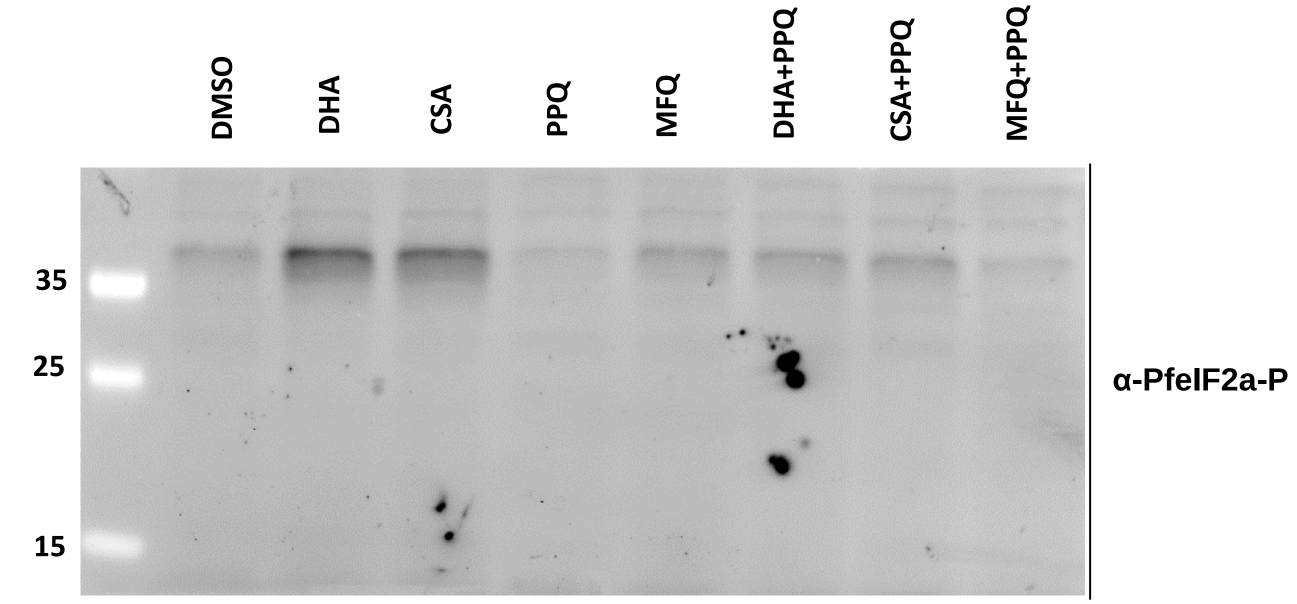

DMSO
DHA
CSA
PPQ
MFQ
DHA+PPQ
CSA+PPQ
MFQ+PPQ
35
25
15
α-PfeIF2a-P

Replicate 3

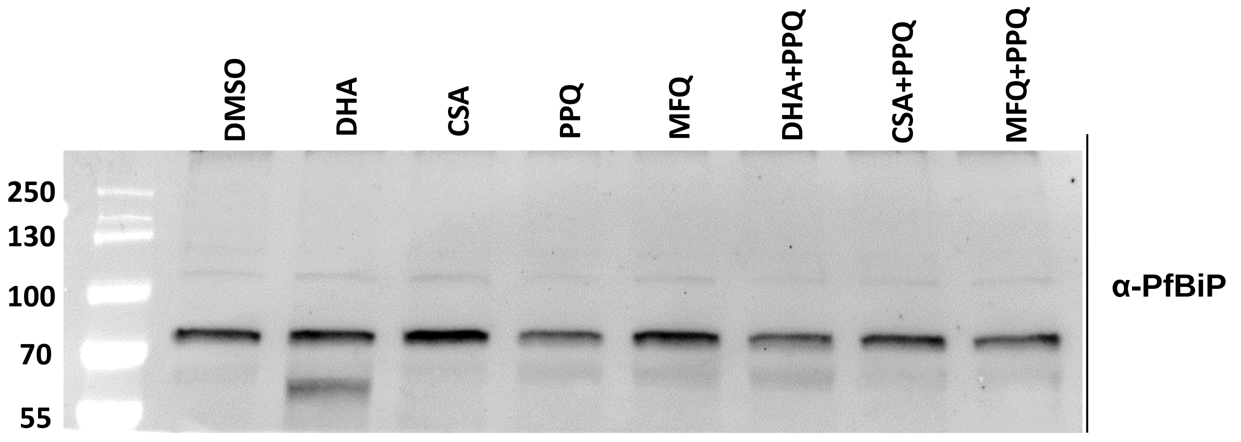
DMSO
DHA
CSA
PPQ
MFQ
DHA+PPQ
CSA+PPQ
MFQ+PPQ
250
130
100
70
55
α-PfBiP

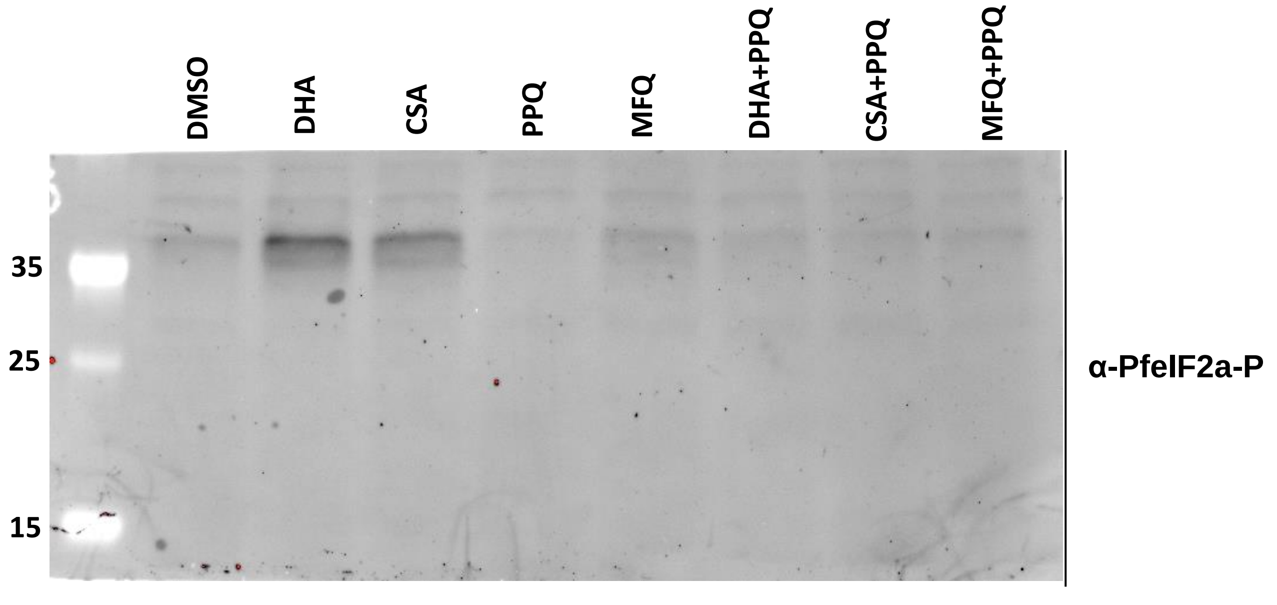
DMSO
DHA
CSA
PPQ
MFQ
DHA+PPQ
CSA+PPQ
MFQ+PPQ
35
25
15
α-PfeIF2a-P

c) Western Blots Fig 2C (right panel)

Replicate 1

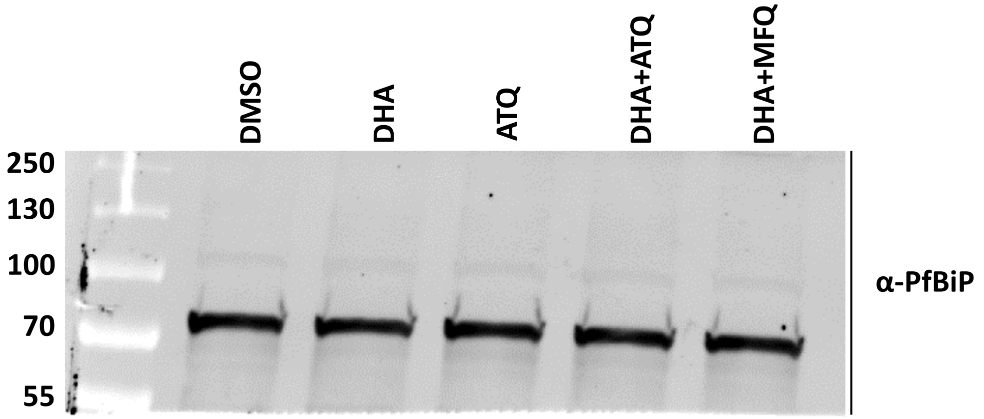

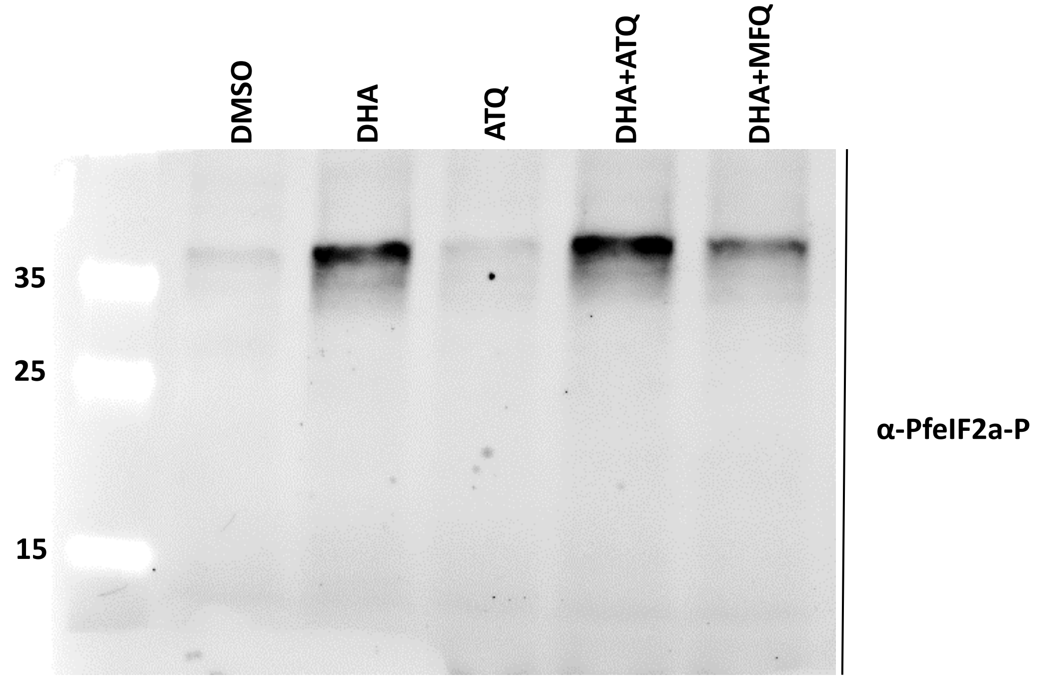

Replicate 2

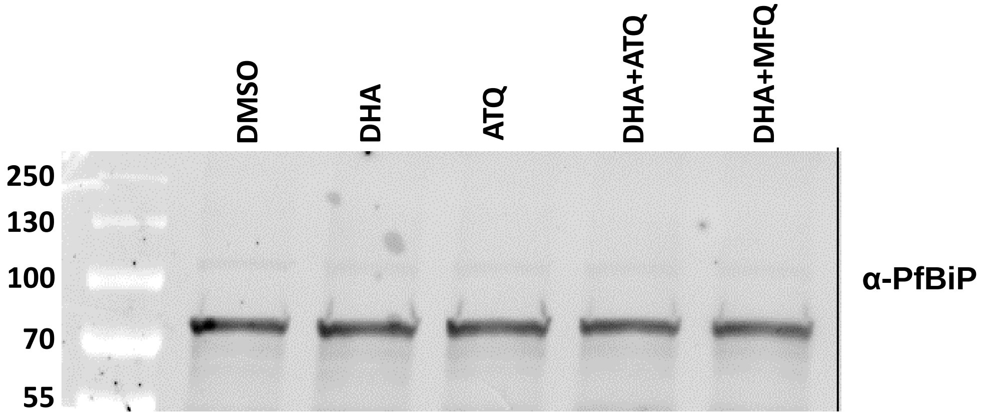
DMSO
DHA
ATQ
DHA+ATQ
DHA+MFQ
250
130
100
70
55
α-PfBiP

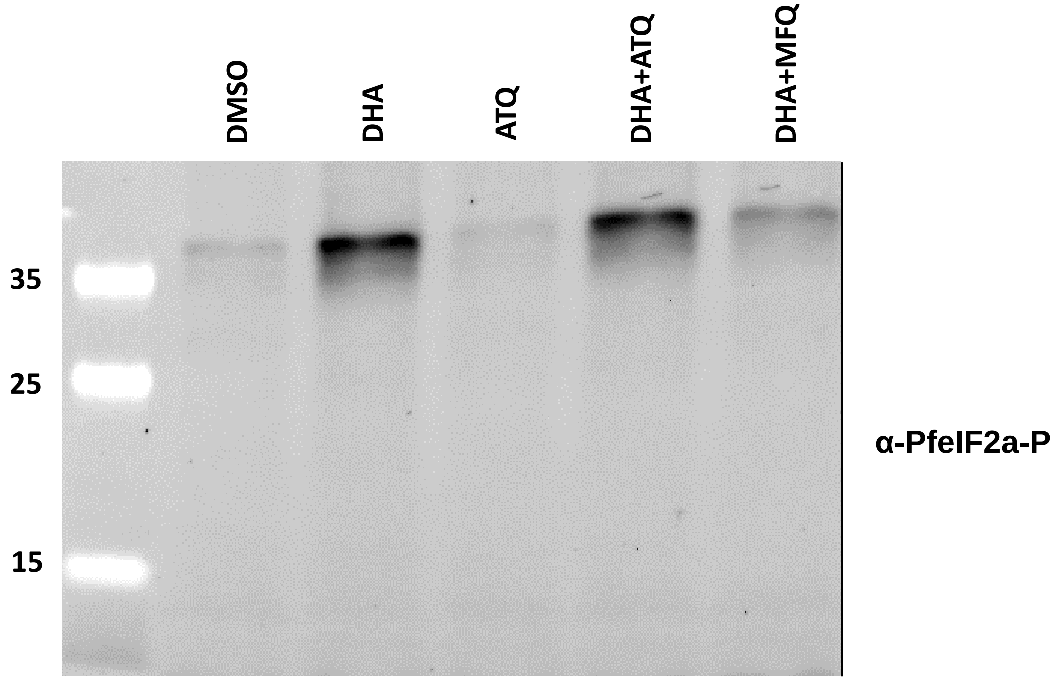
DMSO
DHA
ATQ
DHA+ATQ
DHA+MFQ
35
25
15
α-PfeIF2a-P

Replicate 3

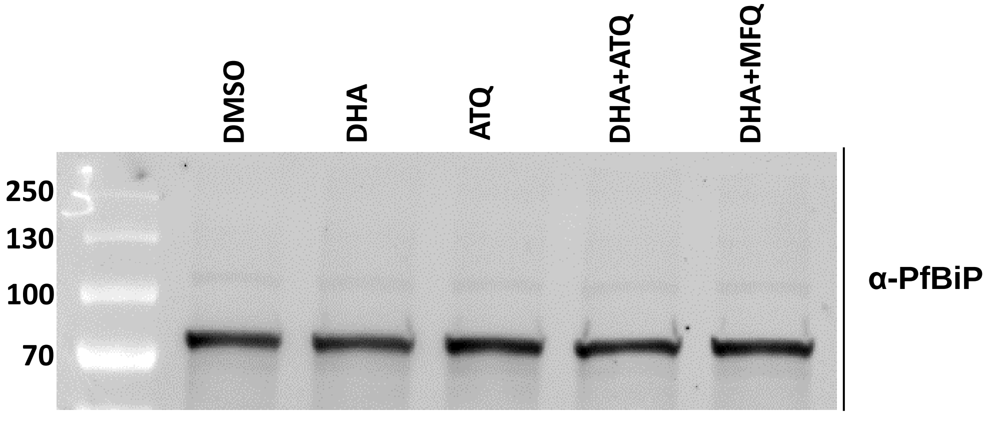
DMSO
DHA
ATQ
DHA+ATQ
DHA+MFQ
250
130
100
70
α-PfBiP

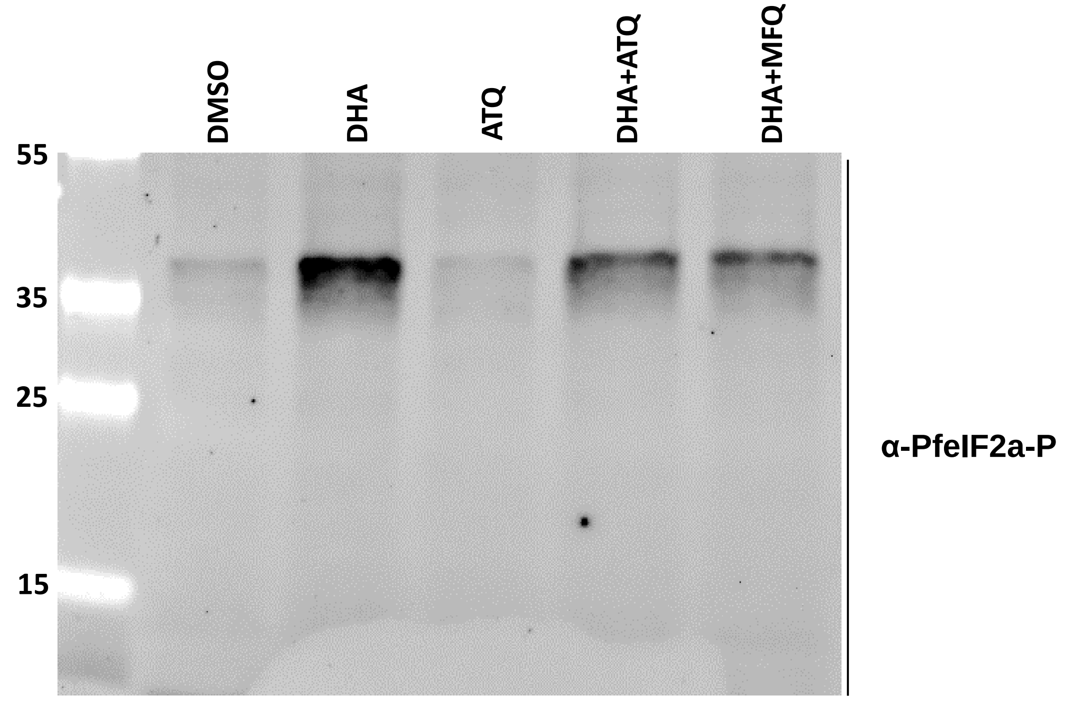
DMSO
DHA
ATQ
DHA+ATQ
DHA+MFQ
55
35
25
15
α-PfeIF2a-P

d) Western Blots Fig 2C (left panel)

Replicate 1

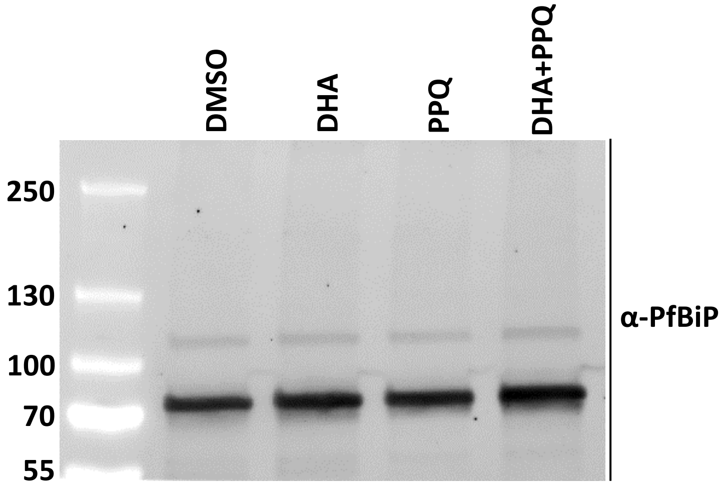

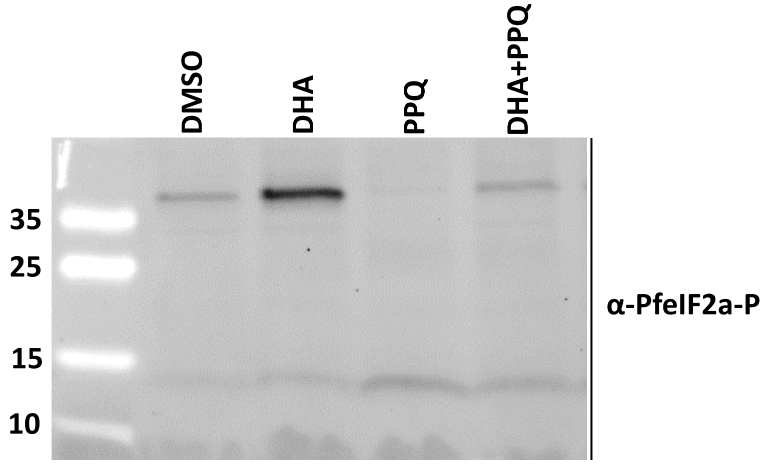

Replicate 2

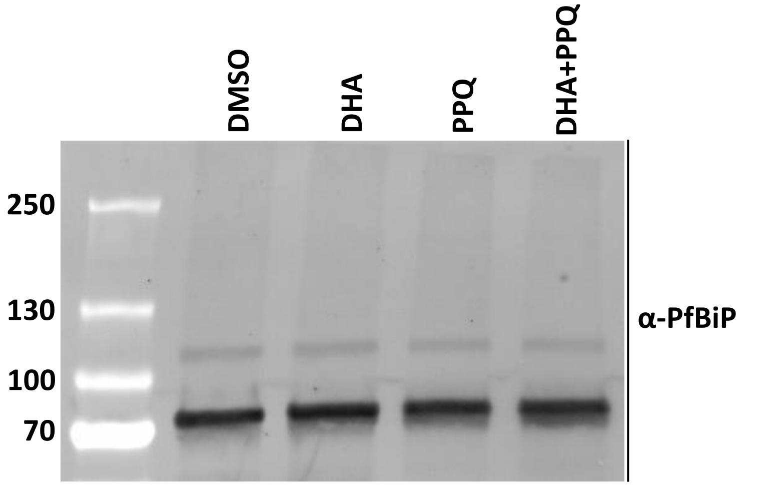
DMSO
DHA
PPQ
DHA+PPQ
250
130
100
70
α-PfBiP

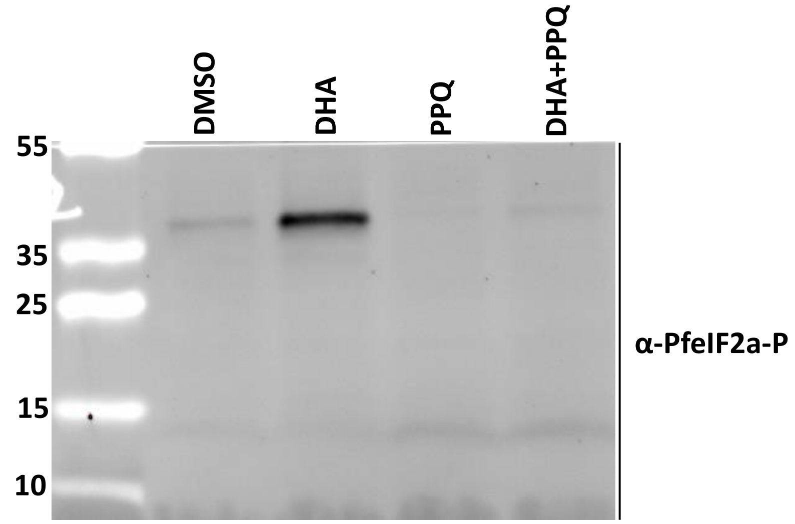
DMSO
DHA
PPQ
DHA+PPQ
55
35
25
15
10
α-PfeIF2a-P

Replicate 3

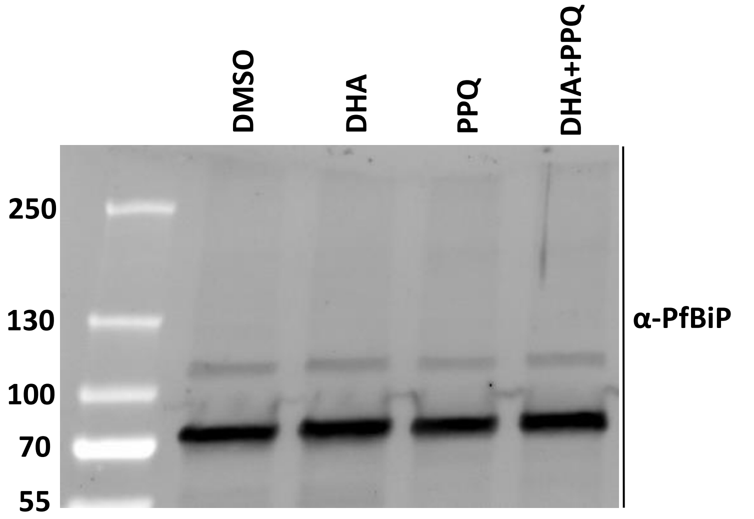
DMSO
DHA
PPQ
DHA+PPQ
250
130
100
70
55
α-PfBiP

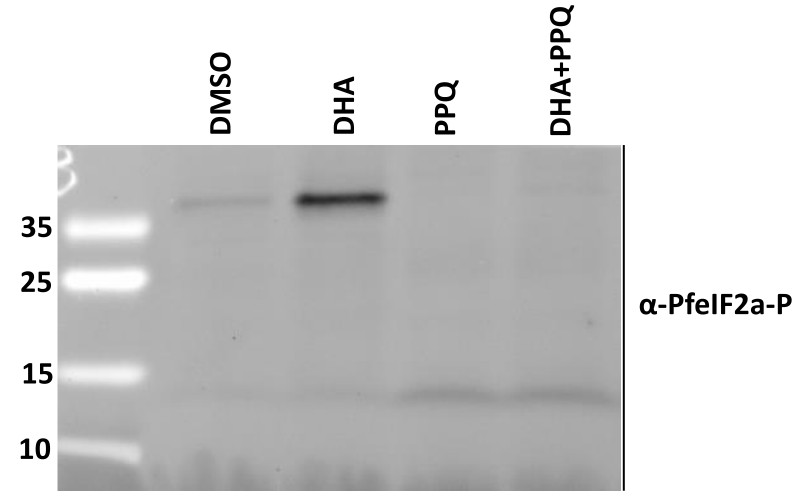
DMSO
DHA
PPQ
DHA+PPQ
35
25
15
10
α-PfeIF2a-P

e) Western Blots Fig 2D

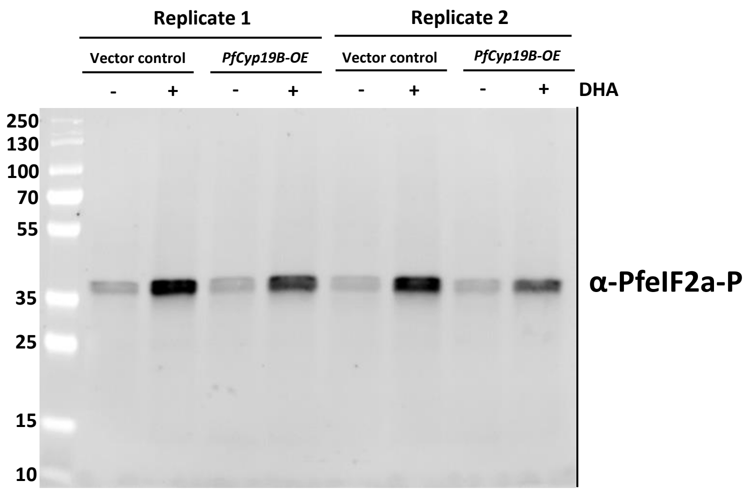

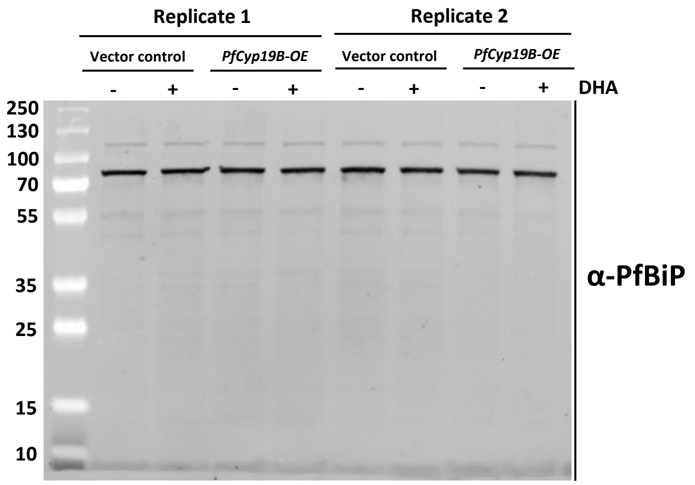

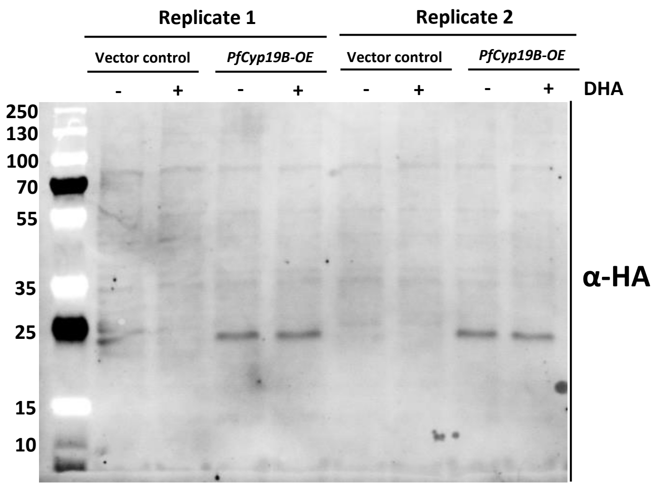

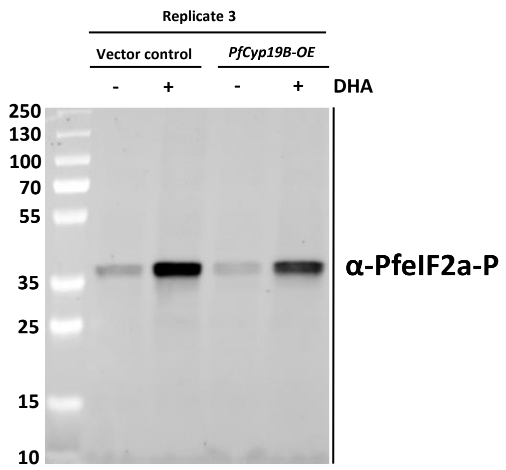
Replicate 3
Vector control
PfCyp19B-OE
- + - + DHA
250
130
100
70
55
35
25
15
10
α-PfeIF2a-P

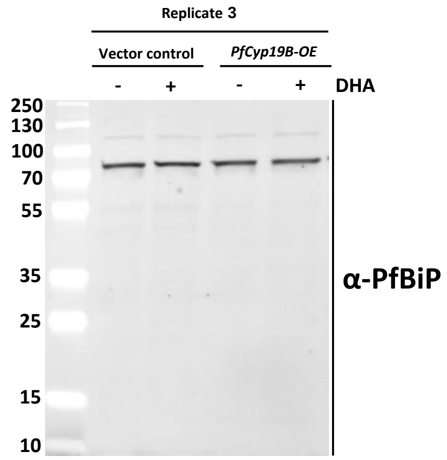
Replicate 3
Vector control
PfCyp19B-OE
- + - + DHA
250
130
100
70
55
35
25
15
10
α-PfBiP

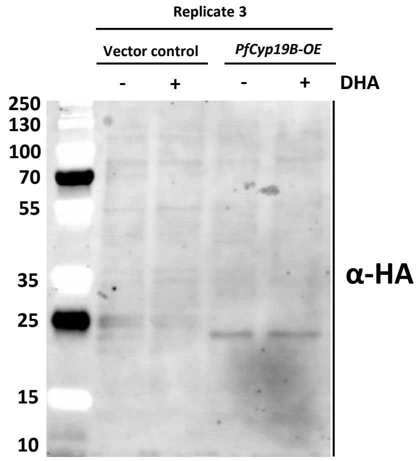
Replicate 3
Vector control
PfCyp19B-OE
- + - + DHA
250
130
100
70
55
35
25
15
10
α-HA

f) Western Blots Fig 2E

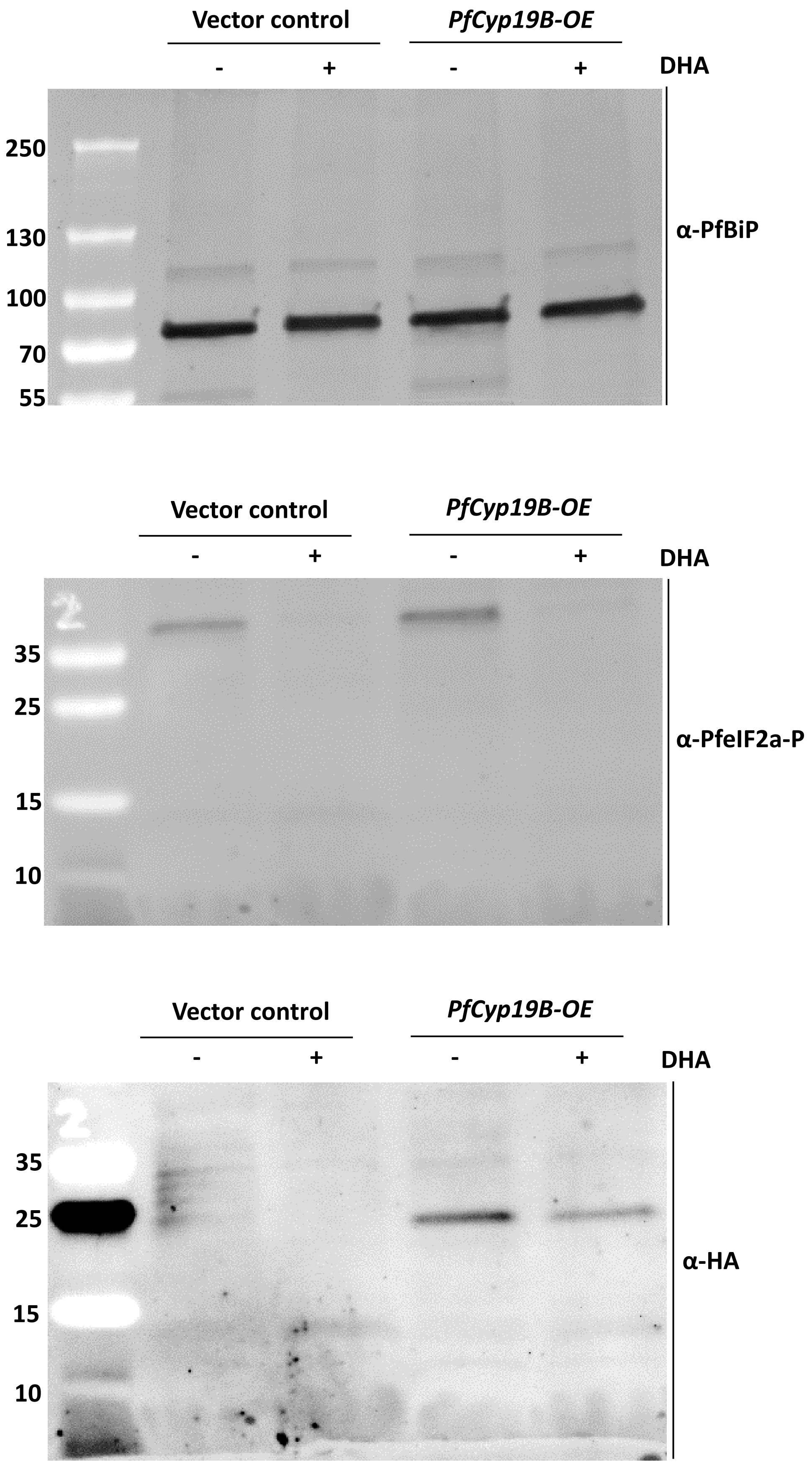

g) Western Blots S4 Fig (gradient)

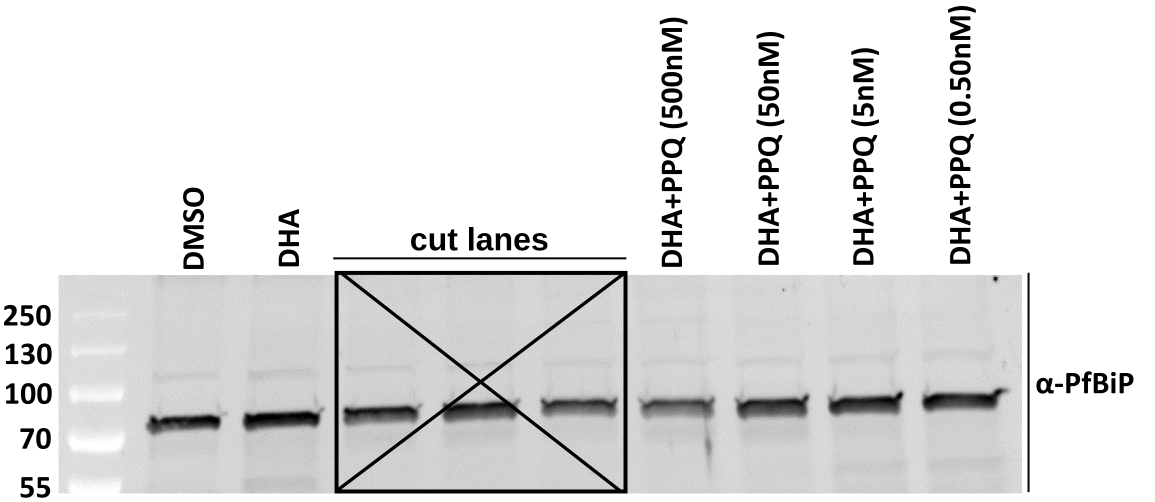

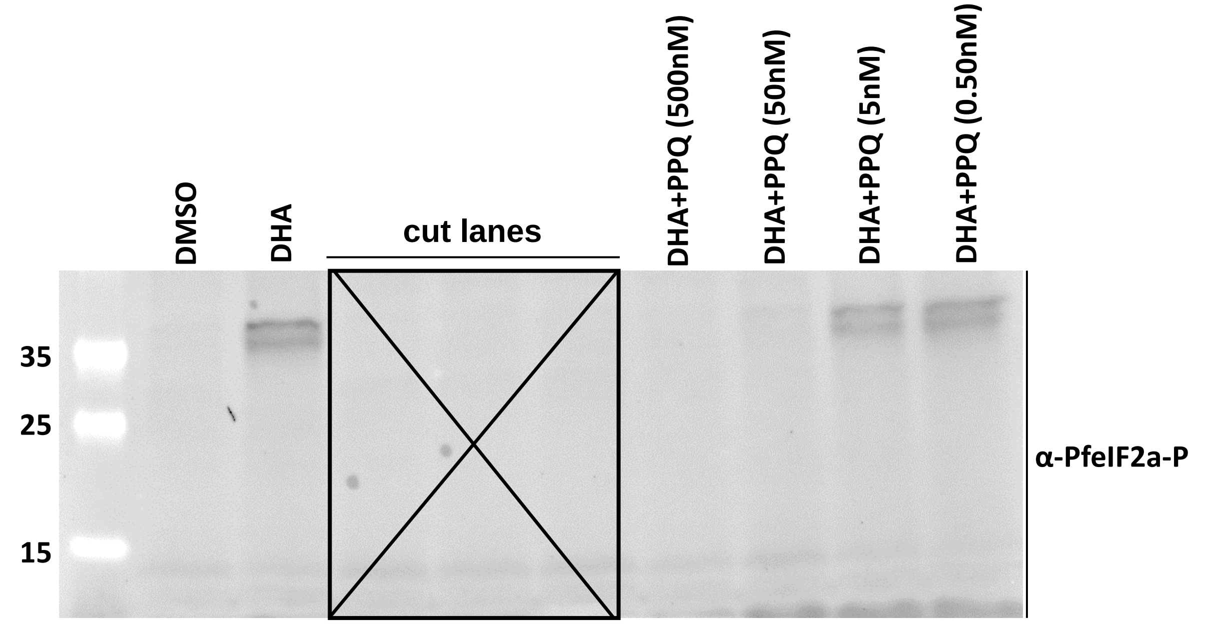

g) Western Blots Fig 3C

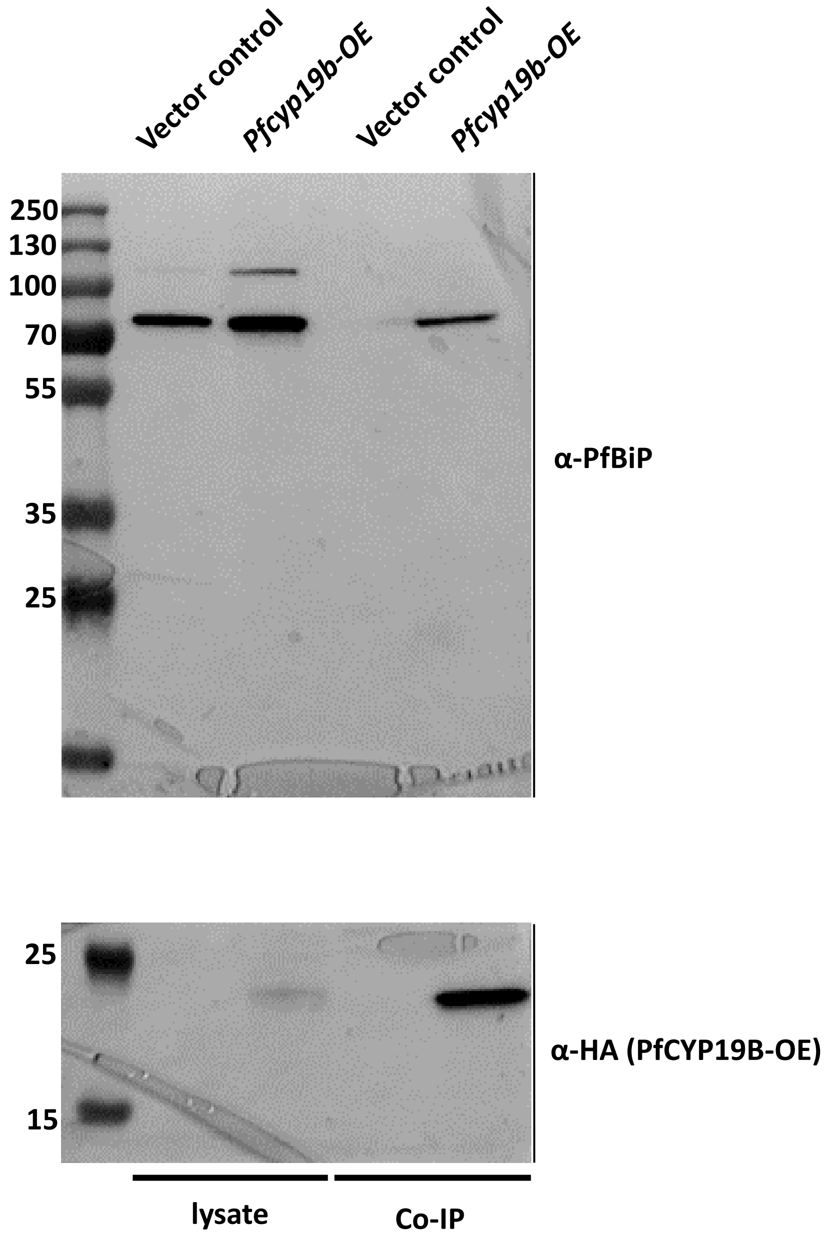

Supplement: S1 Images — Full unprocessed western blot images used in the study. (PDF) [file ppat.1011118.s014.pdf]
